# Integrated Analysis of Whole Exome Sequencing and Copy Number Evaluation in Parkinson’s Disease

**DOI:** 10.1038/s41598-019-40102-x

**Published:** 2019-03-04

**Authors:** Eman Al Yemni, Dorota Monies, Thamer Alkhairallah, Saeed Bohlega, Mohamed Abouelhoda, Amna Magrashi, Abeer Mustafa, Basma AlAbdulaziz, Mohamed Alhamed, Batoul Baz, Ewa Goljan, Renad Albar, Amjad Jabaan, Tariq Faquih, Shazia Subhani, Wafa Ali, Jameela Shinwari, Bashayer Al-Mubarak, Nada Al-Tassan

**Affiliations:** 1 Behavioral Genetics Unit, Department of Genetics, King Faisal Specialist Hospital and Research Centre, P.O Box 3354, Riyadh, 11211 Saudi Arabia; 2 Saudi Human Genome Program, King Abdulaziz City for Science and Technology, P.O Box 6086, Riyadh, 11442 Saudi Arabia; 3 Department of Genetics, King Faisal Specialist Hospital and Research Centre, P.O. Box 3354, Riyadh, 11211 Saudi Arabia; 4 Department of Neurosciences, King Faisal Specialist Hospital and Research Centre, P.O. Box 3354, Riyadh, 11211 Saudi Arabia

## Abstract

Genetic studies of the familial forms of Parkinson’s disease (PD) have identified a number of causative genes with an established role in its pathogenesis. These genes only explain a fraction of the diagnosed cases. The emergence of Next Generation Sequencing (NGS) expanded the scope of rare variants identification in novel PD related genes. In this study we describe whole exome sequencing (WES) genetic findings of 60 PD patients with 125 variants validated in 51 of these cases. We used strict criteria for variant categorization that generated a list of variants in 20 genes. These variants included loss of function and missense changes in 18 genes that were never previously linked to PD (*NOTCH4*, *BCOR, ITM2B*, *HRH4*, *CELSR1*, *SNAP91*, *FAM174A*, *BSN*, *SPG7*, *MAGI2*, *HEPHL1*, *EPRS*, *PUM1*, *CLSTN1*, *PLCB3*, *CLSTN3*, *DNAJB9* and *NEFH*) and 2 genes that were previously associated with PD (*EIF4G1* and *ATP13A2*). These genes either play a critical role in neuronal function and/or have mouse models with disease related phenotypes. We highlight *NOTCH4* as an interesting candidate in which we identified a deleterious truncating and a splice variant in 2 patients. Our combined molecular approach provides a comprehensive strategy applicable for complex genetic disorders.

## Introduction

Parkinson’s disease (PD) is the second most common neurodegenerative disorder associated with a host of motor and non-motor symptoms^1^. These symptoms include muscle rigidity, resting tremor, bradykinesia, and postural instability that maybe accompanied by autonomic dysfunction, sensory symptoms, fatigue, as well as cognitive and behavioral symptoms. Some patients may also develop depression, visual hallucinations, and dementia^2^. A definitive diagnosis is only possible postmortem and is based on the presence of two hallmarks:(1) Lewy bodies in surviving neurons from various brain regions, including the substantia nigra; and (2) the progressive degeneration of the nigrostriatal system^1,3^. Even after many decades of research, PD etiology remains largely unknown. However, research continues to demonstrate the strong genetic component of PD, which is important to understand disease mechanisms^4,5^.

The majority of reported PD cases are sporadic and only 5–10% are regarded as familial. The latter exhibit a monogenic form of the disease with a classical Mendelian mode of inheritance (reviewed in^6^). Despite the rarity of familial PD, family-based studies have been instrumental in identifying at least six genes with confirmed causal link to PD^6–8^. Point mutations, exon deletions, and copy number variants (CNV) in *SNCA* (MIM 163890), *LRRK2* (MIM 609007), *PARKIN* (MIM 602544), *PINK1* (MIM 608309), and *PARK7* (MIM 602533) genes are described in both PD familial and sporadic cases, suggesting that both forms may share the same defective molecular pathways^5,9–15^. While linkage analysis led to the identification of high-penetrance disease-causing mutations, genome wide association studies aided the discovery of lower impact common variants with incomplete penetrance that represent risk factors^16–19^.

Although substantial progress has been achieved in the field of PD genetics, a large portion of the disease burden could not be explained by the identified mutations and risk variants. This is a reflection of the recognized genetic heterogeneity of PD, as allelic variability has been observed not only across populations but also within families^4,13,20–24^. Considering the above, it seems that additional genes have yet to be discovered. This has become more attainable with the emergence of whole exome sequencing (WES) that has been successfully applied in the discovery of novel genes in other complex neurological disorders^25–29^. In PD, WES has led to the identification of more than 40 candidate genes^4,30–34^. At present, supporting functional evidence for a role in PD pathophysiology is available only for three of the candidate genes (*VPS35*,*TMEM230* and *DNAJC13*)^32,35–38^.

Thus far, the majority of WES findings in PD are derived from familial cases, and when possible validated in larger replication cohort with the same form of the disease^4,30,32,34,38^. Such an approach is appealing as rare variants with large effect size tend to aggregate in multi-incident families. However, the more common (sporadic) form of PD remains under-investigated apart from a single attempt in which a large cohort of more than 1000 cases (including non-familial) with early onset PD underwent WES assuming recessive inheritance^33^.

**Figure 1:**
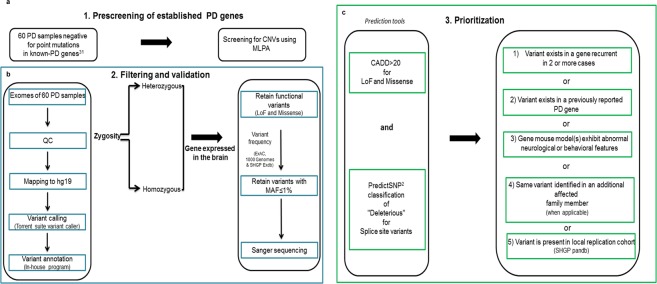
Summary of the 3-stage analysis approach applied in this study. (**a**) Pre-WES mutation screening of reported genes. (**b)** WES filtering and validation. (**c)** Criteria for gene prioritization.

Some analysis strategies for WES data resulted in successful gene discovery in simple Mendelian diseases. However, these strategies are not suited for complex disorders where allelic heterogeneity and oligogenic inheritance are suspected. Here we set out to identify rare variants with likely pathogenic effect representing candidates worthy of further investigation in future studies. To achieve this, we devised a thorough 3-stage analysis strategy to overcome some of the common challenges encountered in gene discovery of complex disorders (Fig. 1). Our stringent prioritization was carried out to increase the likelihood that the candidate variants discovered in this study have a role in PD.

## Results

### *PARKIN* exon 3 deletion

A homozygous deletion of *PARKIN* exon 3 was detected in two early onset cases. One was a familial case (PD-10) with three reported affected siblings, unfortunately; none of the siblings were available for DNA testing. The other case (PD-56) was sporadic with no reported family history of PD. The remaining samples were negative for deletions/duplication in the surveyed genes therefore representing a good sample pool for candidate gene discovery.

### Validated rare variants

As our cohort is a mixture of familial and sporadic cases, we searched for homozygous, compound heterozygous and heterozygous putative variants in all samples regardless of the mode of inheritance and consanguinity. At this stage we focused on rare variants in both local and international frequency databases. This inclusive approach offers a number of advantages (1) exploration of interfamilial and intrafamilial heterogeneity, (2) detection of autosomal recessive variants in seemingly “sporadic” cases with uncertain/unknown family history, (3) minimizing variant filtering flaws (inclusion/exclusion) due to inaccurate/incomplete pedigree information or family history and errors in *in silico* predictions of variant impact.

**Figure 2:**
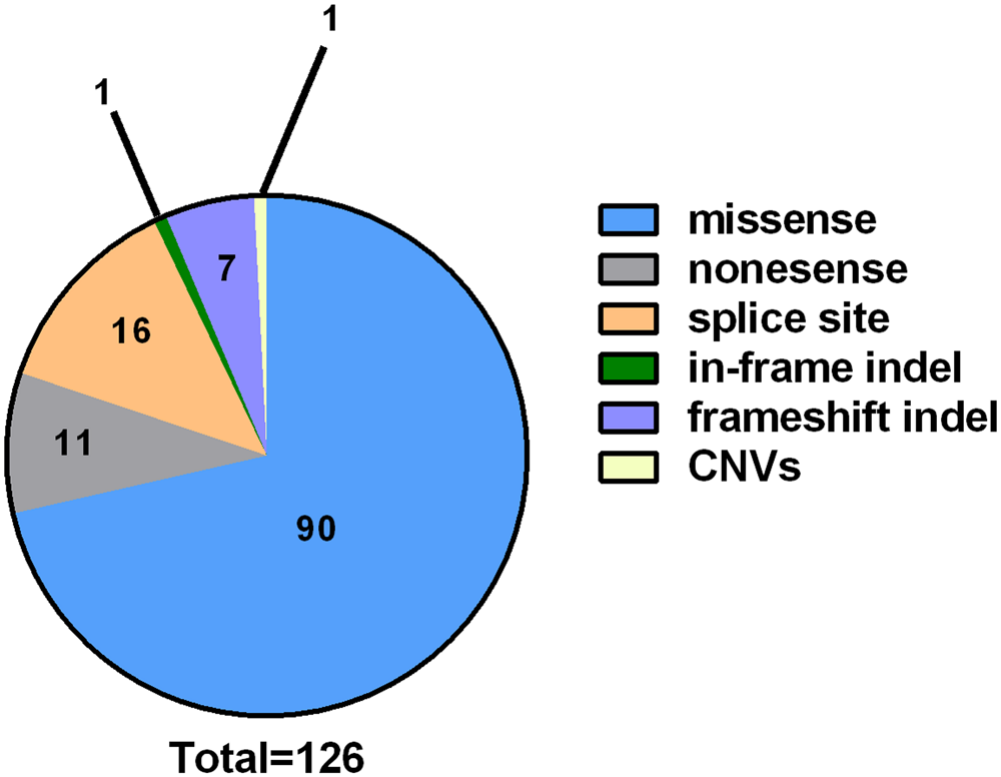
Breakdown of genetic alterations identified in this study. Pie chart illustrating the type and the number of all the validated genetic alterations (SNVs and CNVs) identified in this study.

**Figure 3:**
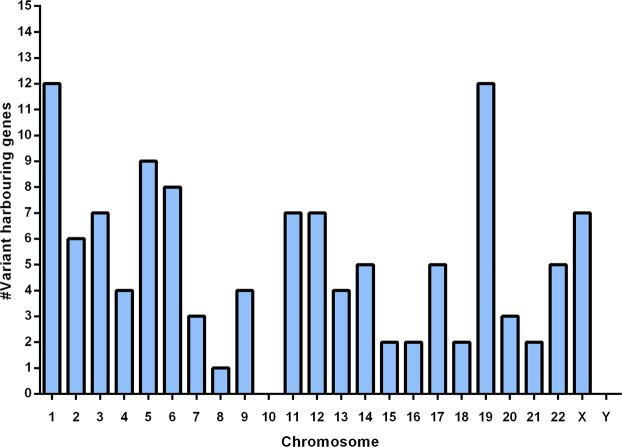
Distribution of variants harbouring genes across the genome. Distribution of the identified genes with validated variants across chromosomes.

Our analysis workflow yielded a total of 125 Sanger validated rare variants in 51/60 (85%) cases with 1–6 variants/sample. Of these variants 90 were missense and 34 were loss of function (LoFs) (Fig. 2). Variants were identified in 117 genes, 13 of which were observed in 2 or more cases (*NOTCH4, BCOR, FAM174A, EIF4G1, DNAJB9, RABEP1, EPRS, BRINP2, HEPHL1, PUM1, GAMT, SH3TC2* and *SEC22A*). Two genes were previously reported in PD (*ATP13A2* and *EIF4G1*)^39–41^ (Fig. 3 and Supplementary Table S1).

Among all the identified variants 39 had a pLI ≥ 0.9, 82 had a positive Z score and 34 had both pLI ≥ 0.9 and a positive Z score. While these gene constraint metrics are useful (in combination with other *in silico* tools) in predicting variant deleteriousness, they are not individually sufficient to infer or exclude pathogenicity^42^. This is supported by the fact that many of the well-established PD genes have high tolerance for missense and/or LoFs (Supplementary Table S2). Therefore, these metrics were neither considered in the filtering process nor included in the subsequent variant prioritization. On the other hand, CADD and PredictSNP^2^ prediction results were taken into account for variant prioritization. It is noteworthy, that none of the pre-screened genes in our cohort^43^ were flagged by our filtering pipeline. This provides reassurance that we did not overlook any candidate or known disease-causing variants in these genes.

In addition to rare variants, we interrogated our PD cohort dataset for shared variants with a deleterious prediction (CADD ≥ 20) these variants were common in the general Saudi population but absent in the international databases. We compiled a list of variants enriched in our cohort and unique to the Saudi population (Supplementary Table S3). Although this list is derived from a small sample, it has the potential to inform variant selection for association studies if empowered with results from a larger cohort in future research.

### Prioritised rare variants

Next, we further restricted our list of candidate variants on the basis of fulfilling at least 1 out of 5 proposed conditions (Fig. 1c). Our aim was to discriminate variants that are most likely to contribute to the phenotype. These variants would represent promising candidates that warrant further functional investigation. This strategy produced a list of 22 prioritized single nucleotide variants (SNVs) in 20 genes (Table 1). Five genes carried multiple variants in unrelated patients. A nonsense variant (p.R151X) in *FAM174A* was detected in two familial cases and their affected relatives (PD-19 and PD-34). Both affected siblings in PD-19 were heterozygous for *PARIKIN* exon3 deletion^43^. The index case in PD-34 was homozygous for this variant while his affected siblings and offspring were heterozygous. This is unsurprising, since inter and intrafamilial differences were previously observed in PD cases^22,23^. A splice-donor variant (c.2591 + 3 G > A) occurring in *PUM1* was present in two familial cases (PD-32 and PD-46). The third recurring variant was a missense (p.K715R) within *EIF4G1* that was found in two sporadic cases (PD-56 and PD-62) and a familial case (PD-23).

**Table 1:** List of Prioritized Variants.

Proband ID/(Gender)	Form of PD/Consanguinity	Identified Variant				Genotype	
		Gene	Base Change	Amino Acid Change	Proband	Other Family Members	Criteria/prioritization
PD-1 (M)	SP/No	*NOTCH4*	c.2865 + 2T > C	—	T/C	NA	1,3,5
PD-2 (M)	SP/No	*BCOR*	c.478G > A	p.V160I	Hemi (A)	NA	5
PD-5 (M)	SP/No	*ITM2B*	c.575A > T	p.Y192F	A/T	NA	3
PD-6 (M)	FM/No	*HRH4*	c.145C > T	p.R49X*	C/T	Father (Aff): C/T	4
PD-13 (M)	SP/No	*CELSR1*	c.8006T > G	p.L2669R	T/G	NA	3
PD-16 (F)	FM/No	*BSN*	c.7808G > A	p.R2603Q	G/A	NA	3
PD-17 (F)	SP/No	*SNAP91*	c.737G > A	p.R246Q	G/A	NA	3
PD-19^a^ (M)	FM /No	*FAM174A*	c.451C > T	p.R151X*	C/T	Brother (Aff): C/T	1,4
PD-23 (M)	FM /No	*EIF4G1*	c.2144A > G	p.K715R	A/G	NA	1,2
PD-28 (M)	NA	*SPG7*	c.1027A > G	p.K343E	G/G	NA	3
PD-29 (M)	SP/No	*MAGI2*	c.1280C > T	p.T427I	C/T	NA	3,5
PD-31 (M)	SP/Yes	*ATP13A2*	c.1544C > T	p.T515M	C/T	NA	2,3
PD-32 (M)	FM/No	*PUM1*	c.2591 + 3G > A	—	G/A	NA	1
PD-33 (M)	SP/Yes	*CLSTN1*	c.553C > G	p.Q185E*	C/G	NA	3,5
PD-34^b^ (M)	FM/No	*FAM174A*	c.451C > T	p.R151X*	T/T	2 affected male siblings (C/T) 2 affected male offspring (C/T)	1,4
PD-40 (M)	SP/No	*PLCB3*	c.698 + 4A > T	NA	A/T	NA	5
PD-43 (M)	SP/No	*EPRS*	c.2513G > A	p.R838H*	G/A	NA	1,3
PD-45 (M)	SP/No	*DNAJB9*	c.626G > A	p.R209Q	G/A	NA	3
PD-46 (F)	FM /No	*PUM1*	c.2591 + 3G > A	—	G/A	NA	1
PD-47 (M)	FM /Yes	*HEPHL1*	c.1856A > G	p.N619S	A/G	Son (Aff): A/G	4
PD-53 (F)	SP/No	*CLSTN3*	c.1124C > T	p.T375I	C/T	NA	3
PD-56^c^ (M)	SP/No	*EIF4G1*	c.2144A > G	p.K715R	A/G	NA	1,2
PD-57 (F)	SP/No	*EPRS*	c.2372A > G	p.Y791C	A/G	NA	1,3
PD-58 (M)	SP/Yes	*NEFH*	c.964C > G	p.R322G	C/G	NA	3
PD-62 (M)	SP/No	*EIF4G1*	c.2144A > G	p.K715R	A/G	NA	1,2
PD-64 (M)	SP/No	*NOTCH4*	c.3769C > T	p.Q1257X	C/T	NA	1,3

SP: Sporadic, FM: Familial, NA: Information not available, or not applicable. Aff: Affected. ^a^A heterozygous deletion of *PARKIN* exon3 was previously reported in this index case and his affected sibling^43^, confirmed by MLPA in this study. ^b^Intrafamilial differences is observed. ^c^A homozygous deletion of *PARKIN* exon3 was identified by MLPA in this study in this sample. *Reported in dbSNP (MAF recorded in Supplementary Table S1). Prioritization based on filtering pipeline summarized in Fig. 1.

Among the recurrent genes was *EPRS*, in which two distinct missense variants (p.R838H and p.Y791C) were detected in two sporadic cases (PD-43 and PD-57), respectively. The former case presented with late onset, while the latter had an early onset form. *NOTCH4*, also harboured two distinct LoF variants (c.2865 + 2T > C and p.Q1257X) in two sporadic cases (PD-1 and PD-64). The former had an early age of onset (41 years) but that of the later was not reported. To our knowledge, none of the shortlisted genes, except for *ATP13A2* and *EIF4G1*, have been previously reported in PD^39–41,44^.

### Biological processes and networks over-represented in our genes set

The assessment of the resulting genes list with Ingenuity Pathway Analysis (IPA) discovered “Cell Death and Survival”, “Cellular Assembly and Organization” and “Cell Morphology” to be among the top ranking molecular and cellular functions. As for system level functions, “Nervous System Development and Function”, “Tissue Morphology” and “Embryonic Development” were among the top 5 (Supplementary Table S4). On the other hand, Gene Ontology (GO) analysis revealed significant enrichment in terms pertaining to the nervous system. Terms like “Nervous system development (GO:0007399)”,“Neuron part (GO:0097458)”, “Postsynaptic density (GO:0014069)”, “Postsynaptic specialization (GO:0099572)”, “Asymmetric synapse (GO:0032279)”, and “Neuron to neuron synapse (GO:0098984)”, were all over-represented (Supplementary Table S5). Both approaches identified categories specific or related to the nervous system. In addition, differential brain expression was also determined from public databases (Supplementary Table S6).

## Discussion

Next Generation Sequencing (NGS) is widely used to identify disease causing variants in monogenic and complex disorders^45–47^. Both exome and targeted sequencing are used as molecular research tools for gene discovery in neurological disorders^25,26,48,49^. The success of WES in identifying causative mutations in different disorders has encouraged its application in clinical settings^26,50^. And with the implementation of precision medicine and through incorporation of molecular screening as part of routine clinical practice, identification of rare variants in complex disorders becomes a research priority.

PD-NGS oriented studies focused on small sets of familial cases or trios, with a few exceptions where large cohorts were used to identify de novo and inherited mutations. Interestingly, the majority of the identified mutations represent private changes restricted to a single family or case, which is expected considering the heterogeneity of the disease^4,33^. These results suggested that infrequent low penetrant mutations in PD patients could be a major cause of the disease^4,51^. Hence, searching for a prevalent causative gene/mutation is unreasonable especially that mutations in some of the previously known PD genes with established disease related functional roles are rare^21,30,32,35^.

Identifying rare variants with minor allele frequencies (MAF) (≤%1) in genes expressed in the brain and/or in molecular pathways linked to a neurological disorder could contribute to our understanding of the genetic basis of PD. We complied a cohort of both familial and sporadic cases that were subjected to WES and analysed the data regardless of consanguinity or mode of inheritance using a strict multistage filtering. The analysis yielded a general list of rare variants in 51/60 cases and prioritized list of potential disease related variants in 25 index cases (Table 1, Supplementary Table S1).

In total 20 genes were shortlisted with potential disease related variants; all these genes were not previously linked to PD except for *EIF4G1* and *ATP13A2*, where there are conflicting reports on their role in familial PD^39,41,44^. Eleven variants were absent in our local control database and 4 out of these were recorded in international databases, the remaining had a MAF of less than 1% in local and/or international databases. Although the majority of the SNVs identified in this study were unique events in a single family or a sporadic case; our pipeline identified multiple variants in 5 genes. Two different variants were detected in 2 of these genes (*NOTCH4* and *EPRS*), whereas *PUM1*, *FAM174A* and *EIF4G1* had a single recurrent variant (Table 1, Supplementary Table S1).

All the cases selected for WES in this study were negative for point mutations and confirmed CNV changes in the known genes^43^, with the exception of one familial and a sporadic case each carried *PARKIN* exon3 deletion and an additional WES identified variant. The two affected siblings from PD-19 were heterozygous for the *PARKIN* exon3 deletion and both had a truncating variant in *FAM174A*, this may represent a case of digenic mechanism for disease progression^52^. The sporadic case PD-56, homozygous for *PARKIN* exon3 deletion, had a heterozygous rare missense variant. Although *PARKIN* exon3 deletion is a confirmed disease-causing mutation^13,53,54^, these additional variants may play a role in disease progression and may contribute to its phenotypic heterogeneity.

Assigning disease causality of newly identified variants requires rigorous functional assessment and segregation analysis, however, when feasible; replication cohorts provide evidence that may support novel findings^4,33^. One of the limitations of our study is the lack of unaffected family members or parents for segregation analysis and since we did not have access to a powerful PD disease-related exome dataset; we further investigated the occurrence of our identified variants in Saudi Human Genome Program (SHGP) patient database, which contained cases with overlapping neurodegenerative phenotypes. We found that 7 of our variants were also present in patients with overlapping phenotypes (Supplementary Table S7), which further supports the role of these genes in the development of PD.

To gain an insight into the impact of the identified genes on the central nervous system (CNS), we surveyed the Mouse Genome Informatics (MGI; http://www.informatics.jax.org) database for any available transgenic mice for our candidate genes with a neurological phenotype^55^. The knockout mouse models of 13 of the prioritized genes presented with different neurological and behavioral phenotypes, including aging related phenotypes, tremors, impaired limb coordination, abnormal gait, abnormal synaptic vascular formation and other specific neurological phenotypes. Of these, *NOTCH4* and *CLSTN3* showed extensive regional brain anomalies. Tremors and/or abnormal balance were present in *SPF7*, *ATP13A2*, *CLSTN3* and *NEFH* transgenic mice, while involuntary movement and limb gasping were observed characteristics in the *CELSR1* and *SNAP91* transgenic mice, respectively (Supplementary Table S8).

In addition to mining mouse models databases, both GO and IPA analysis of our selected genes list have identified significant enrichment of biological processes/terms related to the nervous system and neuronal function (Supplementary Tables S4 and S5). This enrichment further supports the hypothesis that these genes may influence key neuronal functions and contribute to the disorder.

Taken together, from the 18 genes prioritized here, *NOTCH4* is the strongest PD candidate gene with a unique truncation and a splice site variant identified in two sporadic cases; the splice site variant was also present in a familial case in the SHGP pandp (Supplementary Table S7) with an overlapping phenotype. There are conflicting reports about the linkage of *NOTCH4* variants to neurological conditions including schizophrenia and Alzheimer’s disease but none linking it to PD^56–59^. Several Notch4-allele targeted and knockout mouse models were developed, including reporters and inducible transgenics. Interestingly, one of these transgenes, Tg(tetO-Notch4*)1Rwng (MGI:5502689), contains DNA encoding amino acids 1411–1964 of Notch4 placed under the control of the tetracycline response element and is used to model arteriovenous malformations of the human brain (DOID:0060688). This inducible transgenic mouse exhibits multiple brain abnormalities and increased neuron apoptosis. These mice also present with ataxia and seizures^60,61^ (Supplementary Table S8). The Notch hetero-oligomer contains 6 characterized domains; a large extracellular domain (ECD), with 10–36 tandem Epidermal Growth Factor (EFG)-like repeats which participate in ligand interactions; a negative regulatory region, containing three cysteine-rich Lin12-Notch Repeats (LNR); a single transmembrane domain (TM); a small intracellular domain (ICD), which includes a RAM (RBPjk-association module); in addition to six ankyrin repeats (ANK), involved in protein-protein interactions; and a PEST domain^62,63^. The p.Q1257X variant, identified in PD-64 falls in the (LNR) repeat involved in receptor regulation, while the splice variant (c.2865 + 2T > C) affects an exon that falls within Calcium-binding EFG-like domain. It is well established that Notch signalling is an evolutionarily conserved pathway involved in a wide variety of developmental processes, including adult homeostasis, stem cell maintenance, cell proliferation and apoptosis^62,64^.

In summary, we used a combination of gene dosage and NGS analysis to screen for changes in both familial and sporadic PD cases. Using this approach we identified at least a single potential disease related genetic event in 51/60 cases studied. Our strict filtering and prioritizing criteria retained at least one single rare variant in (26/51) 50% of the cases studied; some of these variants are in strong candidate genes with known brain related functions while others may represent low penetrance risk alleles. Failure to identify potential candidate variants in the remaining cases could be attributed to a number of reasons, including missing variants in poorly covered regions or variants in non-coding or regulatory regions. There is also the possibility of gene dosage alterations existing in genes not included in this study. This approach is suitable for a complex heterogeneous disorder with different molecular mechanisms at play.

## Conclusions

We have used a stringent multistage filtering of WES data to identify potential disease related variants in a cohort of both sporadic and familial cases. We identified a number of interesting variants in genes not previously linked to PD. Our data is consistent with previous WES studies where the majority of variants and candidate genes identified represent private events with very low or no rate of replication. The integrative approach employed here generated a useful catalogue of rare potentially deleterious PD candidate variants for further genetic replication and functional assessment studies.

## Methods

### Patients and samples

We assembled a cohort of 60 Saudi patients (19 familial and 41 sporadic) whom all presented with a consultation of PD symptoms (Supplementary Tables S9, S10 and Fig. S1). Pathogenic point mutations in PD major genes were previously ruled out in these patients^43^.

### Multiplex ligation-dependent probe amplification (MLPA)

Gene dosage alterations were assessed using two commercially available MLPA kits: SALSA MLPA probemix P051-D1 and P052-D1 Parkinson (MRC Holland, The Netherlands) as described^65^ (Fig. 1a). Together, the probemixes contained MLPA probes covering all exons of the following PD related genes: *PARKIN, SNCA, PINK1, PARK7, UCLH1*, and *GCH1*, as well as selected exons of *LRRK2, ATP13A2, CAV1*, and *CAV2*. Different probes covering all exons of *PARKIN* were included in both kits permitting cross verification of any detected changes. Rearrangements detected in *PARKIN* were verified using P052-D1 MLPA assay and/or by conventional PCR using primers flanking the deleted exon as previously described^43^.

### Whole exome sequencing, data processing and primary analysis

Whole exome sequencing and subsequent data analysis for all samples were performed as previously described^48^. Briefly, 100 ng of genomic DNA from each sample was sequenced on the Ion Proton platforms using the whole exome AmpliSeq kit (Life Technologies, Carlsbad, CA, USA). A maximum of 17 Gb of DNA sequence was generated for each sequencing run/sample. First, reads were subjected to quality control (QC) checks to eliminate any low quality reads, then were mapped and aligned to UCSC Human reference genome (hg19) (http://genome.ucsc.edu/) using tmap, which is part of the Torrent Suite package. All variants were called using Torrent Suite Variant Caller (Life Technologies, Carlsbad, CA, USA) and annotated with ANNOVAR (http://annovar.openbioinformatics.org).

### Filtering and validation

Extensive genetic and allelic heterogeneity, incomplete penetrance and the possible presence of phenocopies, all have often been observed in complex disorders such as PD^30,33–36^. With this in mind, we decided to filter for variants with homozygous and heterozygous transmission for both familial and sporadic cases- regardless of the observed/predicted mode of inheritance and consanguinity. This is to allow for interfamilial and intrafamilial heterogeneity and to avoid missing autosomal recessive variants in sporadic cases^66,67^. Only genes with positive brain expression in publicly available databases (as listed in Gene cards) were selected. Of the selected genes, only functional variants (LoF and missense) were retained before applying the allele frequency filter. We used a stringent minor allele frequency filter of (MAF ≤ 1%) in the SHGP local “ethnically matching” database and/or international (ExAC and 1000 Genomes)^42,68^. The resulting variants were then validated using Sanger sequencing (Fig. 1b).

### Local databases and controls

The SHGP database constitutes NGS data from exomes and 13 targeted gene panels including a panel specific for neurological disorders^48,69^. At the time of the analysis, the exome database included 2379 local control exomes (termed here SHGP Exdb). The neurological disorders gene panel comprised 1863 patients diagnosed with different neurological disorders including neurodegeneration and neuropathy (this patient database is referred to as SHGP pandb). These databases are from a population with high endogamy and consanguinity and is enriched with rare recessive disease causing mutations, some with founder effect^69^. We used the SHGP Exdb as the ethnically matched controls and the SHGP pandb as our local replication cohort.

### Variant prioritization

Because WES generates a large number of variants even after the initial filtration, we created a list of prioritized variants worthy of further investigation in future studies. It is important to point out that we deliberately avoided using the American College of Medical Genetics (ACMG) variant classification system which is only intended for Mendelian disorders and is considered unsuitable approach for complex disorders^70^. We therefore, have devised a prioritization strategy in which only variants predicted to be strongly deleterious (CADD > 20 and PredictSNP^2^ classification of “deleterious” for splice site variants) were considered. Of note, only splice site variants affecting exons not subject to alternative splicing were considered^71,72^. Candidate variants were further prioritized on the bases of meeting at least 1 of 5 strict criteria (1) presence in a gene that was observed in 2 or more cases, (2) presence in a gene previously associated with PD, (3) the gene harbouring the variant has a mouse model with documented neurological or behavioural deficits, (4) same variant was found in additional affected family members (when available), and finally (5) same variant was observed in our local replication cohort (SHGP pandb) (Fig. 1c).

### IPA and GO-enrichment analysis

To identify the functional categories enriched in our genes set (genes containing variants with CADD > 20 and PredictSNP^2^ classification of “deleterious” for splice site variants), we used two independent web-based applications; Gene Ontology (GO) enrichment analysis (http://geneontology.org/)^73^ and the Ingenuity Pathway Analysis software core analysis function (IPA^®^,v01-08,QIAGEN, Redwood City, www.qiagen.com/ingenuity). Our genes set was analyzed for any significant (p < 0.05 and Bonferroni corrected) over-represented GO terms under the three main categories (molecular function, cellular component and biological process). For the IPA core analysis, we first uploaded the genes accession numbers into the software before running either “Expression” or “Variant effect” core analysis (both gave identical results). The analysis was set using the “Ingenuity Knowledge base” as a reference set. The pre- analysis filtering included all “Data sources”, “Tissues and cell lines” and “Mutations findings from the knowledge base”. IPA uses right-tailed Fisher’s exact test to calculate the statistical significance of the resulting functions, pathways and networks. Only the top 5 terms under each category are listed in this study. As for networks, only those with a score >5 were considered.

### Ethics, consent and permissions

We declare that informed consents were obtained from all participants in adherence with the declaration of Helsinki and according to KFSHRC IRB and Research Advisory Committee (RAC) rules and regulations under the following approved project (RAC# 2110035).

## Supplementary information

Supplementary material

## Data Availability

The data supporting the results of this article are included within the article and its additional files. Additional datasets used and/or analyzed during the current study are available from the corresponding author on request.
