# Supplementary material for: Integrated Analysis of Whole Exome Sequencing and Copy Number Evaluation in Parkinson’s Disease
Source: Sci Rep. 2019 Mar 4;9:3344. doi: 10.1038/s41598-019-40102-x (PMC6399448; doi:10.1038/s41598-019-40102-x)
Supplement: Supplementary file 1 — Supplementary material [file 41598_2019_40102_MOESM1_ESM.pdf]

Integrated Analysis of Whole Exome Sequencing and Copy Number Evaluation in Parkinson's Disease

Eman Al Yemni [1,2], Dorota Monies [2,3], Thamer Alkhairallah [4], Saeed Bohlega [4], Mohamed Abouelhoda [2,3], Amna Magrashi [1], Abeer Mustafa [1], Basma AlAbdulaziz [1,2], Mohamed Alhamed [3], Batoul Baz [1], Ewa Goljan [2,3] , Renad Albar [2,3], Amjad Jabaan [2], Tariq Faquih [2,3], Shazia Subhani [2,3], Wafa Ali [3], Jameela Shinwari [1], Bashayer Al-Mubarak [1,2*], Nada Al Tassan[1,2*].

[1]Behavioral Genetics Unit, Department of Genetics, King Faisal Specialist Hospital and Research Centre. P.O Box 3354, Riyadh 11211, Saudi Arabia
[2]Saudi Human Genome Program, King Abdulaziz City for Science and Technology, P.O Box 6086, Riyadh, 11442, Saudi Arabia
[3]Department of Genetics, King Faisal Specialist Hospital and Research Centre. P.O. Box 3354, Riyadh 11211, Saudi Arabia
[4]Department of Neurosciences, King Faisal Specialist Hospital and Research Centre. P.O. Box 3354, Riyadh 11211, Saudi Arabia
[*]To whom correspondence should be addressed: naltassan@kfshrc.edu.sa (NAT). BAl-Mubarak@kfshrc.edu.sa (BM)

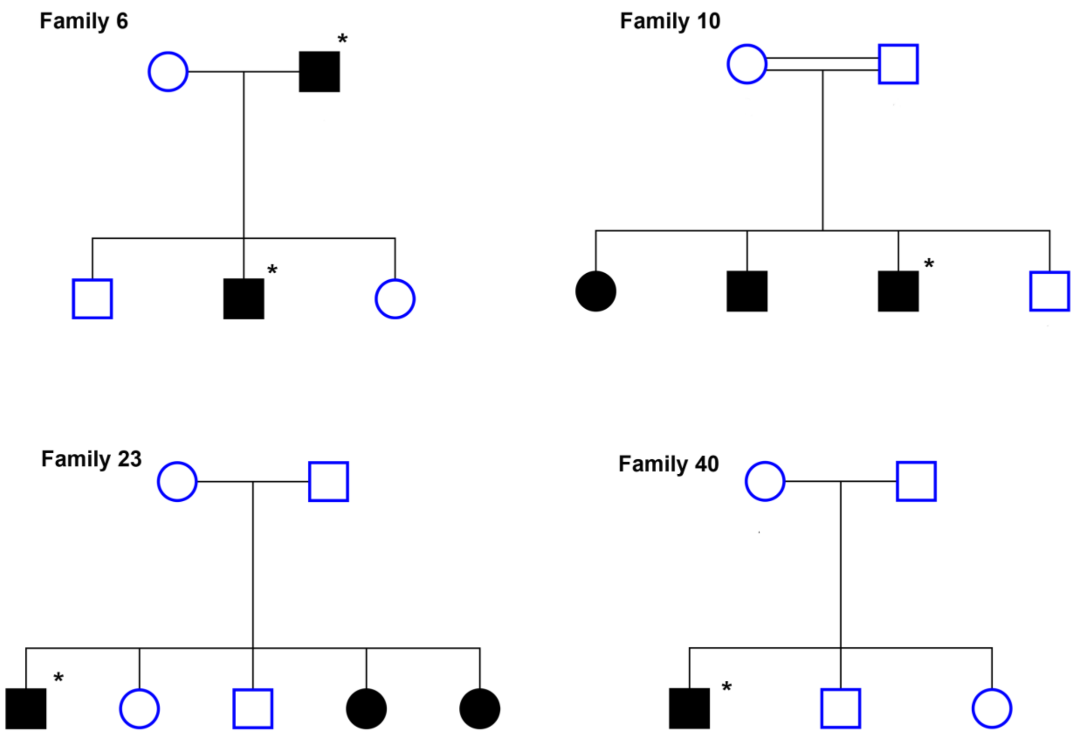

Supplementary Figure 1. Families pedigrees. Example of familial and sporadic cases pedigrees. * indicates the individuals that were enrolled for molecular screening.

Supplementary Table S1. List of all validated SNV's/sample.

			Identified Variant			Genotype		Prediction tools		MAF		Disease database
**Proband ID (Gender)**	**Form of PD / Consanguinity**	**Age of disease onset (years)**	**Gene**	**Base Change**	**Amino Acid Change**	**Proband**	**Other Family Members**	**pLI/ Z score**	**CADD**	**SHGP Exdb**	**ExAC/ 1000 Genomes**	**Human Disease/OMIM**
PD-1 (M)	SP/No	41	*NOTCH4* NM_004557	c.2865+2T>C	_	T/C	NA	0.00/ -0.28	24.4	0	0/0	NA
PD-2 (M)	SP/No	NR	*BCOR* NM_001123383	c.478G>A	p.V160I	Hemi (A)	NA	1.00/ 1.06	25.2	0	0/0	Microphthalmia, syndromic 2 /300166
			SEMA5A NM_003966	c.271-4A>G	_	A/G	NA	0.00/ 1.68	1.533	0.0008	0/0	NA
PD-3 (M)	SP/No	NR	*FBXO38* NM_001271723	c.1327G>C	p.D443H	G/C	NA	1.00/ 1.44	27.4	0	0.00001/0	Neuropathy, distal hereditary motor, type IID/615575
			BRINP2 ^ *NM_021165*	c.656C>T *	p.T219M	C/T	NA	1.00/ 0.79	24.5	0.004	0.00002/0	NA
PD-4 (M)	SP/No	44	*PCDHGA3* NM_018916	c.1426G>A	p.A476T	G/A	NA	NA	24.5	0	0/0	NA
PD-5 (M)	SP/No	41	*CYB5R4* NM_016230	c.445+4A>G	_	A/G	NA	0.00/ 0.46	16	0.0025	0/0	NA
			ITM2B NM_021999	c.575A>T	p.Y192F	A/T	NA	0.72/ 1.04	26.2	0	0/0	Dementia, familial British /176500/117300/ Retinal dystrophy with inner retinal dysfunction and ganglion cell abnormalities/616079
PD-6 (M)	FM/No	37	*HRH4* NM_001160166	c.145C>T	p.R49X	C/T	Father (Aff):C/T	0.00/ -0.32	37	0.0004	0.00002473/0	NA
			GON4L^ NM_001037533	c.6380G>A	p.E2127G	A/G	Father (Aff): A/G	1.00/ 2.42	15	0.0002	0/0	NA
			KDM5A NM_001042603	c.1927A>T *	p.T643S	T/T	Father (Aff):A/T	1.00/ 2.54	17	0	0/0	NA
			DNAH9 NM_001372	c.7013C>T *	p.P2338L	C/T	Father (Aff):C/C	0.00/ 1.94	27.4	0	0/0	NA
			MARS NM_004990	c.2390C>T	p.T797I	C/T	Father (Aff):C/C	0.00/ 0.78	11.45	0.0004	9.561e-05/0.0001	Interstitial lung and liver disease/615486

			TNFAIP3 NM_001270507	c.1811C>G	p.T604R	C/G	Father(Aff):C/C	1.00/1.12	12.32	0.001	1.656e-05/0.0001	Autoinflammatory syndrome, familial, Behcet-like/616744
PD-8 (M)	SP/NA	68	*SGCG* NM_000231	c.623G>T	p.G208V	G/T	NA	0.01/-1.24	25.8	0.00065	0.00003576/0	Muscular dystrophy, limb-girdle, type 2C/253700
PD-10[a] (M)	FM/Yes/ 3 affected siblings	NR	*CLCN7* NM_001287	c.545T>C	p.L182P	T/C	NA	0.98/0.32	16.4	0	0/0	Osteopetrosis, autosomal dominant 2/166600/ Osteopetrosis, autosomal recessive 4/611490
			PTK7 NM_152882	c.1411G>A[*]	p.V471M	G/A	NA	0.98/0.66	18.34	0	0/0	NA
			SNX33 NM_153271	c.134G>A	p.R45H	G/A	NA	0.01/1.82	23.2	0	0.000008252/0	NA
PD-11 (M)	FM/No/1 affected sibling	NR	*ASPM* NM_018136	c.4930A>G[*]	p.R1644G	A/G	NA	0.00/0.11	16.3	0	0/0	Microcephaly 5, primary, autosomal recessive /605481
			PSMD13 NM_175932	c.161_163del insGAG	p.N55D	-/GAG	NA	0.99/0.79	0.038	0.0004	0/0	NA
			GAMT NM_138924	c.790C>T	p.R264X	C/T	NA	0.00/-0.09	11	0.0021	0.0001/0	NA
PD-12 (M)	SP/NA	25	*MAGEB2* NM_002364	c.617C>G	p.P206R	Hemi (G)	NA	0.01/-2.53	19.18	0	0/0	NA
			ST8SIA4 NM_005668	c.297G>C	p.K99N	G/C	NA	0.68/1.51	19.33	0	0/0	NA
			PDE4A NM_001111309	c.38C>T	p.P13L	C/T	NA	0.98/3.22	19.88	0	0/0	NA
PD-13 (M)	SP/No	60	*CELSR1* NM_014246	c.8006T>G	p.L2669R	T/G	NA	1.00/0.18	29.9	0.00042	0/0	NA
PD-15 (M)	SP/No	12	*RABEP1* NM_001083585	c.164-3C>T	_	C/T	NA	0.98/1.17	14.59	0	0/0	NA
PD-16 (F)	FM/No	57	*BSN* NM_003458	c.7808G>A	p.R2603Q	G/A	NA	1.00/0.61	23.1	0	0.00003343/0	NA
			NFASC NM_001160331	c.101C>T	p.P34L	C/T	NA	1.00/2.26	29.9	0	1.678e-05/0	NA
			LRIG1 NM_015541	c.707G>C	p.S236T	G/C	NA	0.84/0.95	21.3	0	3.298e-05/0	NA
			HECTD4 NM_001109662	c.1801C>T[*]	p.R601W	C/T	NA	1.00/6.5	29.1	0.003	0/0	NA
			GRIK5 NM_002088	c.2174T>C	p.M725T	T/C	NA	0.92/3.85	9.5	0	0/0	NA

PD-17 (F)	SP/No	44	*SNAP91* NM_001242792	c.737G>A	p.R246Q	G/A	NA	0.97/ 0.52	27	0	0.00006807/0	NA
			DMRT3 NM_021240	c.821G>A	p.C274Y	G/A	NA	0.00/ 0.86	18.9	0	8.246e-06/0	NA
PD-18 (F)	SP/No	30	*BCOR* NM_001123383	c.1282G>C	p.E428Q	C/A	NA	1.00/ 1.06	8.9	0	0/0	Microphthalmia, syndromic 2/300166
PD-19[b] (M)	FM/No	45	*FAM174A* NM_198507	c.451C>T	p.R151X	C/T	Brother (Aff):C/T	0.00/ 0.48	41	0.0025	0.00002/0	NA
			CSN3 NM_005212	c.433G>A	p.A145T	A/A	WT	0.00/ -0.91	12.99	0.001	0.1/0 **	NA
			TGM2 NM_004613	c.467G>A	p.R156Q	G/A	WT	0.00/ 0.59	15.3	0	5.767e-05/0	NA
			PLCG1 NM_002660	c.1294G>C	p.V432L	G/C	WT	0.76/ 4.46	25.2	0	0/0	NA
			VAMP7 NM_001185183	c.128T>C	p.L43P	Hemi (C)	Brother (Aff): Hemi (C)	0.00/ 0.30	16.6	0	0/0	NA
PD-20 (M) Brother of PD-19 (M)	FM/No	55	*FAM174A* NM_198507	c.451C>T	p.R151X	C/T	Brother (Aff):C/T	0.00/ 0.48	41	0.0025	0.00001663/0	NA
			VAMP7 NM_001185183	c.128T>C	p.L43P	Hemi (C)	Brother (Aff): Hemi (C)	0.00/ 0.30	16.6	0	0/0	NA
			NSMF ^ NM_001130969	c.134-4C>T	_	C/T	WT	0.05/ 2.63	6.751	0.007	0/0	Hypogonadotropic hypogonadism 9 with or without anosmia/614838
			ATG4C NM_032852	c.957delT	p.C319 Wfs*23	T/-	WT	0.00/ -2.20	34	0.002	0/0	NA
PD-21[c] (M)	FM/NA	31	*ADRA2B* NM_000682	c.664C>T	p.R222X	C/T	Son (Aff):C/T	0.00/ 0.13	26.8	0.0004	0/0	Epilepsy, myoclonic, familial adult, 2/607876
			MAP7D3 NM_024597	c.1371C>G	p.S457R	G/G	Son (Aff):C/C	0.00/ -0.86	11.2	0	0.00001/0	NA
			AOX1^ NM_001159	c.106C>T	p.R36X	C/T	Son (Aff):C/C	0.00/ -0.89	16.69	0.0037	1.7e-05/0	NA
PD-23 (M)	FM/No/3 affected siblings	NR	*ATG2B* NM_018036	c.4868C>G	p.P1623 R	C/G	NA	0.00/ -0.02	23.3	0.002	0/0	NA

			EIF4G1 [⊥⊥]<br>NM_004953	c.2144A>G	p.K715R	A/G	NA	1.00/<br>0.29	25.3	0.002	0/0	Parkinson disease<br>18/614251
PD-24<br>(M)	SP/No	51	*DYM*<br>NM_017653	c.1282C>T	p.R428X	C/T	NA	0.00/<br>0.27	40	0	0.00001/0	Smith-McCort dysplasia<br>/607326
			UGT2B10<br>NM_001075	c.901A>T	p.N301Y	T/T	NA	0.00/<br>-2.64	23.2	0	2.477e-<br>05/0	NA
PD-28<br>(M)	NA	NR	*SPG7*<br>NM_003119	c.1027A>G	p.K343E	G/G	NA	0.00/<br>-1.48	25.3	0	0/0	Spastic paraplegia 7,<br>autosomal<br>recessive/607259
			DIAPH3<br>NM_00125837<br>0	c.2042C>T	p.S681F	C/T	NA	0.0 /<br>-0.42	0.551	0.005	0/0	Auditory neuropathy,<br>autosomal dominant, 1/<br>609129
PD-29<br>(M)	SP/No	NR	*MAGI2*<br>NM_012301	c.1280C>T	p.T427I	C/T	NA	0.88/<br>-0.12	27.9	0.002	0/0	NA
			FBXO46<br>NM_00108046<br>9	c.112C>A	p.P38T	A/A	NA	0.88/<br>3.22	22.9	0.007	0.12/0 [**]	NA
PD-30<br>(M)	SP/NA?	>50	*GRIN3B*<br>NM_138690	c.1811C>T	p.T604M	C/T	NA	0.00/<br>-0.61	15.97	0.005	9.962e-<br>05/0	NA
			VRK2<br>NM_00128883<br>9	c.829G>T	p.E277X	T/T	NA	0.00/<br>-2.18	31	0.006	0/0	NA
			PCDHAC1<br>NM_018898	c.760C>T [*]	p.R254X	C/T	NA	0.00/<br>0.61	19.07	0.0007	0/0	NA
PD-31<br>(M)	SP/Yes	38	*ATP13A2* [⊥⊥]<br>NM_022089	c.1544C>T [*]	p.T515M	C/T	NA	0.00/<br>-0.50	32	0	0/0	Kufor-Rakeb<br>syndrome//606693
			USP4<br>NM_199443	c.1063C>G	p.L355V	G/G	NA	0.00/<br>0.14	18.11	0	0/0	NA
			UBE2E2<br>NM_152653	c.215C>A	p.P72H	C/A	NA	0.92/<br>2.41	20.7	0	0/0	NA
			SH3TC2<br>NM_024577	c.2693A>G [*]	p.Y898C	A/G	NA	0.00/<br>-2.20	13.27	0	0/0	Charcot-Marie-Tooth<br>disease, type 4C/601596
			NRCAM<br>NM_00103713<br>2	c.1568A>G	p.N529S	A/G	NA	0.46/<br>0.81	24.7	0	0/0	NA
			FBXO22 [^]<br>*NM_147188*	c.1206_1209<br>delTAAA	p.K403N<br>fs*3	TAAA/-	NA	0.89/<br>1.34	34	0.0029	0.0003/0	NA
PD-32<br>(M)	FM/No/1<br>affected sibling	48	*PUM1*<br>NM_014676	c.2591+3G><br>A	–	G/A	NA	1.00/<br>-0.03	14.1	0.0008	0.0002/0	Spinocerebellar ataxia 47/<br>617931

			TRIM32 NM_001099679	c.1632delC	p.H544Tfs*11	C/-	NA	0.10/ -0.92	35	0	0/0	Muscular dystrophy, limb-girdle, type 2H/254110
PD-33 (M)	SP/Yes	>50	*DNAJB9* NM_012328	c.229C>T	p.L77F	C/T	NA	0.22/ 0.50	17.9	0.005	0.00004978/0	NA
			NELL2 NM_001145108	c.122C>T	p.S41F	T/T	NA	1.00/ -0.10	16.02	0	0/0	NA
			USP9X NM_001039590	c.1920A>C	p.Q640H	Hemi (C)	NA	1.00/ 6.35	14.33	0	0.0011/0.0018	Mental retardation, X-linked 99 /300919
			GAS7 NM_003644	c.106-4A>G	_	A/G	NA	0.03/ 3.00	11	0.0004	8.336e-05/0	NA
			CLSTN1 NM_014944	c.553C>G	p.Q185E	C/G	NA	0.84/ 1.13	32	0.0004	0.00014/0	NA
			GLRB NM_000824	c.881C>T*	p.A294V	C/T	NA	0.03/ 0.11	20.7	0	0/0	Hyperekplexia 2/614619
PD-34 (M)	FM/No/ 2 Brothers(Aff)/ 2 Ofs(Aff)	NR	*FAM174A* NM_198507	c.451C>T	p.R151X	T/T	2 Brothers (Aff):C/T , 2 Ofs(Aff) :C/T	0.00/ 0.48	41	0.0025	0.00002/0	NA
			BRINP2 ^ NM_021165	c.656C>T*	p.T219M	T/T	1 Brother (Aff):C/T 1 Brother (Aff):T/T 2 Ofs(Aff): C/T	1.00/ 0.79	24.5	0.004	0.00002/0	NA
			PCNT NM_006031	c.7577C>G	p.A2526G	C/G	2 Brothers (Aff):C/C ,2 Ofs (Aff): C/G	0.00/ -2.01	20.4	0.0057	5.477e-05/0	Microcephalic osteodysplastic primordial dwarfism, type II/210720
PD-35 (M)	SP/No	43	*PDIA6* NM_005742	c.898delG	p.A300Lfs*22	G/-	NA	0.12/ -0.18	35	0	0.00002/0	NA
			SZT2 NM_015284	c.2074C>T*	p.R692C	C/T	NA	0.00/ 2.69	33	0	0/0	Epileptic encephalopathy, early infantile, 18/615476

			ZNF259/ ZPR1 NM_003904	c.350T>G *	p.V117G	T/G	NA	0.02/ 0.83	29	0	0/0	NA
PD-37 (M)	SP/No	35	*FBXO34* NM_017943	c.3_4insCAC C	p. L3Pfs*3 4	CACC/-	NA	0.00/ -0.10	28.4	0	0/0	NA
			ATF6 NM_007348	c.1346A>G	p.K449R	A/G	NA	0.00/ -0.17	23.3	0	0/0	Achromatopsia 7/616517
			CAT NM_001752	c.256G>A	p.E86K	G/A	NA	0.00/ -0.37	36	0.0002	0/0	NA
			RABEP1 NM_00108358 5	c.988_990del AAT *	p.N330K fs*500	AAT/-	NA	0.98/ 1.27	8.318	0	0/0	NA
			USE1 NM_018467	c.257C>A	p.A86D	C/A	NA	0.01/ 0.82	32	0.003	0.00001 /0	NA
			BACE2 NM_138991	c.1465_1467 delCTG	p.L492d el	CTG/-	NA	0.95/ 2.09	15.15	0	0.0003/0	NA
PD-39 (M)	FM/Yes/1 affected sibling	55	*AFMID* NM_00101098 2	c.103C>T	p.R35X	C/T	NA	0.00/ -1.96	22	0.003	0.000033 61/0	NA
PD-40 (M)	SP/No	42	*PLCB3* NM_000932	c.698+4A>T	_	A/T	NA	0.97/- 0.77	10.5	0.0004	0/0	NA
			GAMT NM_138924	c.790C>T	p.R264X	C/T	NA	0.00/ -0.09	11	0.0021	0.0001/0	Cerebral creatine deficiency syndrome 2 /612736
PD-41 (M)	SP/No	NR	*PLCZ1* NM_033123	c.949+3A>G	_	A/G	NA	0.00/ -2.17	0.89	0.0004	0/0	Spermatogenic failure 17/ 617214
PD-42 (M)	SP/No	58	*NGEF* NM_019850	c.990-4G>A	_	G/A	NA	0.95/ 2.18	7.585	0	4.216e-05/0	NA
PD-43 (M)	SP/No	60	*EPRS* NM_004446	c.2513G>A	p.R838H	G/A	NA	0.00/ 0.22	35	0.001	0.0002/0. 00019	Leukodystrophy, hypomyelinating, 15/ 617951
			ZFYVE26 NM_015346	c.2332+3A>T	_	A/T	NA	0.00/ 0.00	14.08	0.0004	0/0	Spastic paraplegia 15, autosomal recessive/270700
PD-44 (M)	SP/No	NR	*SEC22A* NM_012430	c.368A>G	p.K123R	A/T	NA	0.64/ -0.34	28.2	0.0004	0/0	NA
			DOCK6 NM_020812	c.427C>T *	p.R143 W	C/T	NA	0.0/0. 27	14.16	0.001	0/0	Adams-Oliver syndrome 2/614219
PD-45 (M)	SP/No	>50	*DNAJB9* NM_012328	c.626G>A	p.R209Q	G/A	NA	0.22/ 0.50	31	0	0.00002/0	NA
			LRR1 NM_152329	c.338G>A	p.G113D	G/A	NA	0.00/ -0.24	13.78	0	0/0	NA

			SRCIN1 NM_025248	c.2048G>A	p.R683H	G/A	NA	1.00/ 4.43	34	0.026	4.394e-05/0	NA
PD-46 (F)	FM/No	40	*PUM1* NM_014676	c.2591+3G>A	–	G/A	NA	1.00/ -0.03	14.1	0.0008	0.0002/0	Spinocerebellar ataxia 47/ 617931
			LAMC3 NM_006059	c.2189G>C	p.G730A	G/C	NA	0.00/ 0.26	19.4	0	0/0	Cortical malformations, occipital/614115
PD-47 (M)	FM/Yes	NR	*HEPHL1* NM_001098672	c.1856A>G *	p.N619S	A/G	Son(Aff) :A/G	0.00/ -0.10	28.9	0.002	0/0	NA
			APOBEC3C NM_014508	c.403C>A *	p.L135I	C/A	Son(Aff) :C/A	0.09/ -0.08	14.93	0.002	0/0	NA
PD-48 (F)	SP/No	38	*DTNB* NM_001256304	c.1214T>C	p.I405T	T/C	NA	0.00/ -0.47	29.5	0.0004	0.0000141/ 0	NA
PD-52 (F)	SP/NA	70	*SLC22A1* NM_003057	c.280G>T	p.D94Y	G/T	NA	0.00/ 0.53	27.2	0.0003	0/0	NA
			PROM1 NM_006017	c.880G>A	p.V294M	G/A	NA	0.00/ -2.67	15.58	0.0023	0.00002/0	612095/612657/603786/608051/ Macular dystrophy, retinal, 2
PD-53 (F)	SP/No	NR	*CLSTN3* NM_014718	c.1124C>T	p.T375I	C/T	NA	1.00/ -1.13	29.9	0	0.00001/0	NA
			CSNK2A1 NM_001895	c.427-5C>G	–	C/G	NA	1.00/ 3.89	6.002	0	0/0	Okur-Chung neurodevelopmental syndrome/617062
PD-56[d] (M)	SP/No	38	*EIF4G1* ⊥⊥ NM_004953	c.2144A>G	p.K715R	A/G	NA	1.00/ 0.29	25.3	0.002	0/0	Parkinson disease 18/614251
			HEPHL1 NM_001098672	c.751A>G	N251D	G/G	NA	0.00/ 0.10	28.9	0.002	0.02/0 **	NA
PD-57 (F)	SP/No	41	*EPRS* NM_004446	c.2372A>G	p.Y791C	A/G	NA	0.00/ 0.22	25.6	0.002	0/0	Leukodystrophy, hypomyelinating, 15/ 617951
			CHD8 NM_020920	c.6346-5C>T	–	C/T	NA	1.00/ 5.54	1.291	0.00035	4.684e-05/0	Autism, susceptibility to, 18/ 615032
PD-58 (M)	SP/Yes	10	*NEFH* NM_021076	c.964C>G	p.R322G	C/G	NA	0.00/ 0.20	25.5	0	0/0	Amyotrophic lateral sclerosis, susceptibility to/105400
PD-60 (M)	SP/No	>50	*SH3TC2* NM_024577	c.1948G>A	p.E650K	G/A	NA	0.00/ -2.20	15.29	0	2.485e-05/0	Charcot-Marie-Tooth disease, type 4C/ 601596

			HFE NM_000410	c.121G>A	p.D41N	G/A	NA	0.00/ 0.39	13.65	0	0/0	Porphyria cutanea tarda, susceptibility to/176100
			SPTBN2 NM_006946	c.G3431G>A	p.R1144Q	G/A	NA	1.00/ 4.53	12.85	0.0019	5.162e-05/0.00019	Spinocerebellar ataxia 5/ 600224
			ALOX5AP NM_001629	c.353T>G	p.I118S	T/G	NA	0.85/ 0.77	20.7	0.002	0/0	Stroke, susceptibility to/ 601367
			ZNF160 NM_033288	c.760G>A	p.G254R	G/A	NA	0.00/ 0.45	22.9	0	0/0	NA
PD-62 (M)	SP/No	32	*EIF4G1* ⊥⊥ NM_004953	c.2144A>G	p.K715R	A/G	NA	1.00/ 0.29	25.3	0.002	0/0	Parkinson disease 18/614251
			GYS1 NM_001161587	c.1423G>A	p.E475K	A/A	NA	0.01/ 2.41	32	0.0009	0.001/0.0003	NA
			TRPM4 NM_001195227	c.689C>T	p.S230L	T/T	NA	0.00/ 1.46	34	0.001	0.00004/0.001	Progressive familial heart block, type IB/604559
PD-63 (M)	SP/No	>50	*GSTA1* NM_145740	c.276_277insT	p.D93X	-/T	NA	0.06/ -3.79	24.4	0.0004	8.272e-06/0	NA
			TSEN2 NM_025265	c.1087G>A	p.G363R	G/A	NA	0.00/ -1.61	25.6	0	2.471e-05/ 0	Pontocerebellar hypoplasia type 2B/612389
			PCDHB8 NM_019120	c.1092_1093delTG	p.V365Gfs*10	TG/-	NA	0.0/0.93	27.7	0.0004	0/0	NA
PD-64 (M)	SP/No	NR	*NOTCH4* NM_004557	c.3769C>T	p.Q1257X	C/T	NA	0.00/ -0.28	41	0	0/0	NA
			NCF4 NM_000631	c.342+1G>T	_	T/T	NA	0.00/ 0.06	19.65	0	0/0	NA
			ATG10 NM_031482	c.503C>T	p.T168M	C/T	NA	0.00/ -0.27	19.37	0.0008	0.00003/0	NA
			ARHGEF10 NM_014629	c.388C>T	p.P130S	C/T	NA	0.00/ -2.80	19.83	0.0021	8.24e-06/0	Slowed nerve conduction velocity, AD/608236
			XAB2 NM_020196	c.1438G>A	p.V480M	G/A	NA	1.00/ 3.10	18.17	0.0042	0.00002/0	NA
			TENM1 NM_014253	c.5959C>G	p.Q1987E	Hemi (G)	NA	1.00/ 3.29	22.4	0.0012	0/0	NA
PD-76 (M)	SP/No	>50	*STX10* NM_003765	c.206-4C>G	_	C/G	NA	0.00/ 0.73	12.47	0	0/0	NA
			GSTM3 NM_000849	c.272-3C>T	_	C/T	NA	0.00/ 0.03	15.8	0.0016	0/0	NA
			SEC22A NM_012430	c.233A>C	p.Y78S	A/C	NA	0.64/ -0.34	21.7	0.01	0.00002/0	NA

			LRP1 NM_002332	c.6518G>A	p.R2173 Q	G/A	NA	1.00/ 10.62	15.85	0	0.0001/0	NA
PD-78 (M)	SP/No	>50	*PRICKLE3* NM_006150	c.813G>T	p.W271 C	Hemi (T)	NA	0.15/ 0.91	31	0	0/0	NA
PD-79 (M)	SP/No	>50	*ATG16L2* NM_033388	c.565C>T	p.R189C	C/T	NA	0.00/ 0.40	34	0.001	0.0003/0	NA

^ Recorded in homozygous state in SHGP pandp.
⊥⊥ PD associated gene.
* Recorded in other databases with a MAF <1%.
** MAF is above 1% in international databases but less than 1% in local. These variants did not meet the prioritization criteria.
[a] A homozygous deletion of *PARKIN* (exon 3) was identified by MLPA in this study. Affected siblings were not available for segregation analysis
[b] A heterozygous deletion of *PARKIN* (exon 3) was previously reported in this index case and his affected sibling[1], confirmed by MLPA in this study.
[c] A homozygous and heterozygous deletions of *PARKIN* (exon 3) was previously reported in this index case and his son, respectively[1] ,confirmed by MLPA in this study. No clinical data is available for the son; there is growing evidence that heterozygous mutations (including exon 3 deletion) are associated with increased susceptibility to late-onset PD[2].
[d] A homozygous deletion of *PARKIN* (exon 3) was identified by MLPA in this study.
pLI: Intolerance of loss-of-function variation, a pLI score of ≥ 0.9 considered extremely intolerant. Z score: Intolerance of missense or synonymous variation. Z score>0 indicates increased constrain. Z score<0 indicates decreased tolerance.
FM: Familial, SP: Sporadic. Aff: Affected, M: Male, F: Female, Offspring: Ofs, NR: Not reported. NA: Information not available. MAF's were recorded from databases as per date of submission/revision of this manuscript.

Supplementary Table S2. Constraint metrics for PD-established genes (intolerance to variation scores).

Gene	*pLI*	*Z score*
LRRK2	0.0	-0.36
SNCA	0.84	0.54
PARKIN	0.02	-1.1
PINK	0.0	0.08
DJ1	0.9	0.19
VPS35	0.99	3.09
FBXO7	0.10	-0.93
TMEM230	0.01	0.86
DNAJC6	1.00	0.85
DNAJC13	1.00	1.04

In red: PD established genes tolerant to variation.

Supplementary Table S3. Shared variants with CADD above 20.

Identified Variant			Prediction tools		MAF		Disease database	
**Gene**	**Base Change**	**Amino Acid Change**	***pLI/Z* score**	**CADD**	**SHGP NC Database**	**ExAC/1000 Genomes**	**ClinVar**	**Human Disease/OMIM**
QSOX1	c.1479insC	p.S493fs	0.00/ -0.46	22.1	2.372735116	0/0	NA	NA
ATP2B4	c.1372A>C	p.N458H	0.52/2.24	26	1.898188093	0/0	NA	Susceptibility to malaria/611162
DISC1	c.731A>G	p.E244G	0.02/ -0.50	21.6	1.855047455	0/0	NA	Disrupted in Schizophrenia 1/604906
MYOM3	c.1583A>C	p.D528A	0.00/ -1.23	23.1	2.329594478	0/0	NA	NA
ZMPSTE24	c.574G>T	p.G192W	0.00/0.10	33	0.955538222	0/0	NA	Mandibuloacral dysplasia with type B lipodystrophy/608612
NEURL1	c.1414_1415insCG	p. L473Pfs*32	0.54/3.48	22.9	1.553062985	0/0	NA	NA
MXI1	c.13C>G	p.R5G	0.71/1.77	22.3	2.113891286	0/0	NA	Susceptibility to Prostate cancer/176807
TACC2	c.674C>A	p.T225N	0.00/ -1.28	23.3	1.639344262	0/0	NA	NA
ARNTL	c.1472C>A	p.A491E	0.99/3.08	34	1.19176598	0/0	NA	NA
CATSPER1	c.1954G>C	p.V652L	0.00/ 1.18	22.1	0.904977376	0/0	NA	Spermatogenic failure 7/612997
INPPL1	c.2003G>C	p.R668P	0.01/ 1.87	34	1.843317972	0/0	NA	Opsismodysplasia/ 258480
TUBA3C	c.121_122insCA	p. I42Pfs*52	0.01/ 2.51	24.3	1.207937877	0/0	NA	NA
WARS	c.910G>C	p.G304R	0.99/1.65	34	2.070750647	0/0	NA	Neurodevelopmental disorder, mitochondrial, with abnormal movements and lactic acidosis, with or without seizures/617721
CPNE6	c.1244T>C	p.I415T	0.92/2.74	26.8	2.459016393	0/0	NA	NA
CCDC88C	c.5693C>G	p.P1898R	0.00/ -0.51	24	0.774507634	0/0	NA	Spinocerebellar ataxia 40 /616053
BTBD7	c.35G>C	p.C12S	0.68/0.07	26.8	1.42364107	0/0	NA	NA
CATSPER2	c.1439A>G	p.E480G	0.00/1.00	23.8	1.686548745	0/0	NA	Deafness and male infertility/ 611102

SNX22	c.185A>C	p.D62A	0.01/0.04	28.9	1.380500431	0/0	NA	Leukemia, myeloid/ lymphoid or mixed-lineage
NPRL3	c.1020C>A	p.F340L	0.47/0.37	23	4.788610871	0/0	NA	Epilepsy, familial focal with variable foci 3/617118
DNAH3	c.1144C>T	p.P382S	0.00/ -1.74	24.4	2.243313201	0/0	NA	NA
SLC5A11	c.540_541insGC	p. I181Afs*2	0.00/ -0.07	23.2	0.819672131	0/0	NA	NA
PRSS53	c.1435G>T	p.G479W	0.00/ -1.01	34	1.725625539	0/0	NA	NA
RBFOX1	c.392A>G	p.E131G	0.94/0.48	25.8	2.152499088	0/0	NA	NA
CDYL2	c.445_446insTC	p. K150*	0.62/ -0.05	22.5	1.121656601	0/0	NA	NA
TMEM94/KIAA0195	c.2170delC	p.P724fs	0.00/2.42	33	0.9922346	0/0	NA	NA
FHOD3	c.1857insG	p.V619fs	1.00/0.30	27	2.329594478	0/0	NA	NA
SKA1	c.611insA	p.D204fs	0.00/0.46	25.4	2.459016393	0/0	NA	NA
FCHO1	c.1258G>T	p.E420X	1.00/1.96	47	1.941328732	0/0	NA	NA
KLK9	c.617G>C	p.G206A	NA	28.3	1.42364107	0/0	NA	NA
SCN9A	c.1156A>T	p.I386F	0.00/ -0.69	32	1.068200493	0/0	NA	Epilepsy, generalized, with febrile seizures plus, type 7/ 613863
COL5A2	c.2878G>C	p.G960R	1.00/1.11	26.5	1.164596273	0/0	NA	Ehlers-Danlos syndrome, classic type/ 130010
MARCH4	c.20G>T	p.G7V	0.02/1.87	23.6	2.027610009	0/0	NA	NA
COL4A4	c.2090C>T	p.P697L	0.00/ -0.30	23.6	1.766784452	0/0	NA	Alport syndrome, autosomal recessive/ 203780
SP110	c.1801C>T	p.P601S	0.00/ -0.28	23.1	0.888888889	0/0	NA	Hepatic venoocclusive disease with immunodeficiency/ 235550
COL6A3	c.6611G>A	p.G2204E	0.00/ -0.30	22.8	1.527900797	0/0	NA	Bethlem myopathy 1/ 158810
ZNF133	c.1495G>A	p.G499R	0.95/1.50	28.1	1.466781708	0/0	NA	NA
KIAA0930	c.745C>A	p.Q249K	0.82/1.82	21.9	1.98446937	0/0	NA	NA
TRMU	c.229C>T	p.P77S	0.00/ -0.59	28	1.008695652	1.651e-05/0	NA	Liver failure, transient infantile/613070
TAGLN3	c.487T>C	p.S163P	0.69/2.02	24	1.121656601	0/0	NA	NA
CASR	c.1630C>T	p.R544X	0.37/3.52	36	1.869436202	0/0	NA	Hypercalciuric hypercalcemia/601199

ADCY5	c.952G>C	p.G318R	0.99/5.68	25.5	0.677139762	0/0	NA	Dyskinesia, familial, with facial myokymia/606703
COPG1	c.1979T>G	p.V660G	1.00/1.72	26.6	1.898188093	0/0	NA	NA
ENTPD3	c.547G>A	p.G183R	0.00/ -1.01	29.8	2.113891286	0/0	NA	NA
TRAK1	c.2687T>C	p.L896P	0.00/0.92	23.9	2.200172563	0/0	NA	NA
ZMYND10	c.725T>C	p.V242A	0.00/ -0.02	22.6	2.415875755	0/0	NA	Ciliary dyskinesia, primary, 22/615444
BANK1	c.1442C>A	p.P481H	0.00/ -2.21	28.8	1.121656601	0/0	NA	NA
SMAD1	c.175G>A	p.G59R	0.87/2.64	27.8	2.717860224	0/0	NA	NA
DOCK2	c.4064T>C	p.F1355S	1.00/3.80	32	1.251078516	0/0	NA	Immunodeficiency 40/616433
MDC1	c.2555T>G	p.L852X	0.00/0.47	35	0.690250216	0/0	NA	NA
COL11A2	c.524T>C	p.L175P	1.00/2.04	25.3	0.541471819	0/0	NA	Deafness, autosomal dominant 13/120290
HMGA1	c.263A>G	p.K88R	0.72/2.03	22.8	2.113891286	0/0	NA	Diabetes mellitus, noninsulin-dependent, susceptibility/25853
ZAN	c.596G>C	p.R199P	0.00/ -2.00	24	2.717860224	0/0	NA	NA
EPHB6	c.1765C>G	p.P589A	0.25/1.32	26.5	0.733390854	0/0	NA	NA
FKBP6	c.296delC	p.P99fs	0.13/1.52	27.7	1.855047455	0/0	NA	Williams-Beuren syndrome/194050
BLK	c.739C>G	p.L247V	0.00/ -2.17	24.7	2.673796791	0/0	NA	Maturity-onset diabetes of the young, type 11/613375
ASAP1	c.2236T>C	p.S746P	1.00/2.76	25.6	1.294219154	0/0	NA	NA
ZNF462	c.1238C>A	p.A413E	1.00/3.22	26.4	2.761000863	0/0	NA	NA
WDR34	c.142_143insTC	p.P49Cfs*76	0.00/ -0.54	32	0.690250216	0/0	NA	Short-rib thoracic dysplasia 11 with or without polydactyly/615633
TSC1	c.2983_2985delinsCTC	p.C995L	1.00/2.87	21.6	0.050087653	0/0	NA	Lymphangioleiomyomatosis
MAN1B1	c.383_386delinsAAAG	p.L128*	0.00/ -1.01	29.1	0.467451524	0/0	NA	Mental retardation, autosomal recessive 15/614202
IL11RA	c.1165C>G	p.L389V	0.00/0.30	23.2	1.566820276	0/0	NA	Craniosynostosis and dental anomalies/614188
DNAJB5	c.495C>A	p.D165E	0.49/3.42	24.1	2.631578947	0/0	NA	NA
GAB3	c.1424_1425insTG	p. S476Afs*44	0.99/0.86	28.5	1.251078516	0/0	NA	NA
RP2	c.832C>T	p.Q278X	0.85/0.57	39	0.890811911	0/0	NA	Retinitis pigmentosa 2/312600

OTUD5	c.1531G>C	p.A511P	0.98/4.52	23.4	1.466781708	0/0	NA	NA
WNK3	c.1399-2A>-		1.00/1.57	23.9	1.725625539	0/0	NA	NA

Variants are present in the SHGP database with MAF (0.050087653-4.788610871)
This list was generated from a provisional list of 1700 variants after applying quality control checks and omitting variants present in international DNA databases or having a CADD<20.

Supplementary Table S4. Top categories from IPA analysis.

Category	p-value	Molecules	#Molecules
**Top molecular and Cellular Functions**			
Cell Morphology	2.31E-02 - 1.75E-05	24	ATF6,ATP13A2,BSN,CAT,CELSR1,CLSTN1,DNAJB9,EIF4G1,ITM2B,MAGI2,NEFH,NFASC, NOTCH4,NRCAM,NSMF,PCNT,PLCG1,SNAP91,SPG7,SRCIN1,SNX33,TRPM4,ZPR1,ZFYVE26
Cell Death and Survival	2.51E-02 - 1.79E-05	27	ATF6,ATP13A2,CAT,CELSR1,CHD8,CYB5R4,DNAJB9,EIF4G1,GSTA1,ITM2B,LRIG1,MAGI2, NEFH, NOTCH4,NRCAM,NSMF,PCDHGA3,PLCG1,SGCG,SNX33,SPG7,SRCIN1,TRIM32,TRPM4,USE1, ZFYVE26,ZPR1
Cellular Compromise	2.63E-02 - 1.79E-05	15	ATF6,ATP13A2,CAT,CYB5R4,CSPG7,DNAJB9,GSTA1,ITM2B,NEFH,NFASC,PCNT,SNX33,TRPM4, ZFYVE26,ZPR1
Cellular Assembly and Organization	2.63E-02 - 6.31E-05	28	ATG2B,ATG4C,ATF6,ATP13A2,BSN,CAT,CELSR1,CLSTN1,CLSTN3,DYM,EIF4G1,ITM2B,MAGI2, NEFH,NFASC,NRCAM,NSMF,PCDHB8,PCDHGA3,PCNT,PLCG1,SNAP91,SNX33,SPG7,SRCIN1, USE1,ZFYVE26,ZPR1
Cellular Development	2.63E-02 - 6.31E-05	19	ATF6,BCOR,BSN,CAT,CELSR1,CLSTN1,CLSTN3,MAGI2,NEFH,NOTCH4,NRCAM,NSMF, PCDHB8, SNAP91,SRCIN1,TENM1,TRIM32ZFYVE26,ZPR1
**Top Physiological System Development and Function**			
Tissue Morphology	2.31E-02 - 1.79E-05	21	ATP13A2,BSN,CAT,CHD8,CLSTN1,CLSTN3,MAGI2,NEFH,NFASC,NOTCH4,NRCAM,NSMF, PCDHGA3,PDIA6,PLCB3,SNAP91,SPG7,SRCIN1,TRPM4,ZFYVE26,ZPR1
Embryonic Development	2.63E-02 - 6.31E-05	18	ADRA2B,ATF6,BCOR,BSN,CAT,CELSR1,CHD8,CLSTN1,LRIG1,MAGI2,NEFH,NOTCH4,NRCAM, SRCIN1 ,PLCB3,PLCG1,SZT2,ZPR1
Nervous System Development and Function	2.63E-02 - 6.31E-05	25	ADRA2B,ATF6,ATP13A2,BSN,CAT,CELSR1,CLSTN1,CLSTN3,GLRB,GSTM3ITM2B,MAGI2, NEFH, NFASC,NRCAM,NSMF,PCDHB8,PCDHGA3,SNAP91,SPG7,SRCIN1,SZT2,TENM1,ZFYVE26,ZPR1

Organismal Development	2.63E-02 - 6.31E-05	24	ADRA2B,ALOX5AP,ATF6,BCOR,BSN,CAT,CELSR1,CHD8,CLSTN1,CLSTN3,DNAJB9,ITM2B, LRIG1,MAGI2,NEFH,NOTCH4,NRCAM,NSMF,PLCB3,SGCG,SNAP91,SRCIN1,SZT2 ,ZPR1
Tissue Development	2.63E-02 - 6.31E-05	24	
**Top Diseases and disorders**			
Cancer	2.63E-02 - 8.41E-06	69	ADRA2B,AFMID,ALOX5AP,ATF6,ATG16L2,ATG2B,ATG4C,ATP13A2,BCOR,BRINP2,BSN,CAT, CELSR1,CHD8,CLSTN1,CLSTN3,CYB5R4,DNAH9,DNAJB9,DTNB,DYM,EIF4G1,EPRS,FAM174A, FBXO22,FBXO34,FBXO38,FBXO46,GLRB,GSTA1,GSTM3,GYS1,HEPHL1,HRH4,ITM2B,LRIG1, MAGI2,NCF4,NEFH,NFASC,NOTCH4,NRCAM,NSMF,PCDHB8,PCDHGA3,PCNT,PDIA6,PLCB3, PLCG1, PRICKLE3,RABEP1,SEC22A,SGCG,SLC22A1,SNAP91,SNX33,SPG7,SRCIN1,SZT2,TENM1,TRIM32, TRPM4,TSEN2,UBE2E2,UGT2B10,USE1,VRK2,ZFYVE26,ZNF160
Gastrointestinal Disease	2.63E-02 - 8.41E-06	69	ADRA2B,AFMID,ALOX5AP,ATF6,ATG16L2,ATG2B,ATG4C,ATP13A2,BCOR,BRINP2,BSN,CAT, CELSR1,CHD8,CLSTN1,CLSTN3,CYB5R4,DNAH9,DNAJB9,DTNB,DYM,EIF4G1,EPRS,FAM174A, FBXO22,FBXO34,FBXO38,FBXO46,GLRB,GSTA1,GSTM3,GYS1,HEPHL1,HRH4,ITM2B,LRIG1, MAGI2,NCF4,NEFH,NFASC,NOTCH4,NRCAM,NSMF,PCDHB8,PCDHGA3,PCNT,PDIA6,PLCB3, PLCG1, PRICKLE3,RABEP1,SEC22A,SGCG,SLC22A1,SNAP91,SNX33,SPG7,SRCIN1,SZT2,TENM1,TRIM32, TRPM4,TSEN2,UBE2E2,UGT2B10,USE1,VRK2,ZFYVE26,ZNF160
Hepatic System Disease	2.63E-02 - 8.41E-06	56	ADRA2B,ATF6,ATG16L2,ATG2B,ATG4C,ATP13A2,BCOR,BRINP2,BSN,CAT,CELSR1,CHD8, CLSTN1,CLSTN3,CYB5R4,DNAH9,DNAJB9,DTNB,DYM,EIF4G1,EPRS,FAM174A,FBXO22, FBXO38,GSTM3, GYS1,HEPHL1,HRH4,ITM2B,MAGI2,NCF4,NEFH,NFASC,NOTCH4,NRCAM,NSMF,PCDHB8, PCDHGA3,PCNT,PLCG1,PRICKLE3,SEC22A,SGCG,SLC22A1,SNAP91,SNX33,SPG7,SZT2,TENM1, TRIM32,TRPM4,UBE2E2,UGT2B10,VRK2,ZFYVE26,ZNF160

Organismal Injury and Abnormalities	2.63E-02 - 8.41E-06	70	ADRA2B,AFMID,ALOX5AP,ATF6,ATG16L2,ATG2B,ATG4C,ATP13A2,BCOR,BRINP2,BSN,CAT, CELSR1,CHD8,CLSTN1,CLSTN3,CYB5R4,DNAH9,DNAJB9,DTNB,DYM,EIF4G1,EPRS,FAM174A, FBXO22,FBXO34,FBXO38,FBXO46,GLRB,GSTA1,GSTM3,GYS1,HEPHL1,HRH4,ITM2B,LRIG1, MAGI2,NCF4,NEFH,NFASC,NOTCH4,NRCAM,NSMF,PCDHB8,PCDHGA3,PCNT,PDIA6,PLCB3, PLCG1, PRICKLE3,RABEP1,SEC22A,SGCG,SLC22A1,SNAP91,SNX33,SPG7,SRCIN1,SZT2,TENM1,TRIM32, TRPM4,TSEN2,UBE2E2,UGT2B10,USE1,VRK2,ZFYVE26,ZNF160,ZPR1
Reproductive System Disease	2.16E-02 - 9.74E-06	50	ADRA2B,ALOX5AP,ATF6,ATG2B,ATG4C,ATP13A2,BCOR,BSN,CELSR1,CHD8,CLSTN1,CLSTN3, DNAH9,DNAJB9,DYM,EIF4G1,EPRS,FBXO22,FBXO34,FBXO38,GLRB,GSTA1,HEPHL1,HRH4, LRIG1,MAGI2,NCF4,NEFH,NFASC,NOTCH4,NRCAM,NSMF,PCDHB8,PCDHGA3,PCNT,PLCB3, PLCG1,PRICKLE3,RABEP1,SLC22A1,SNAP91,SNX33,SRCIN1,SZT2,TENM1,TSEN2,UGT2B10, ZFYVE26,ZNF160,ZPR1
**Top Canonical Pathways**	**p-value**	**Overlap**	**Molecules**
Unfolded-protein response	8.61E-04	3/56	ATF6,DNAJB9,PDIA6
D-myo-inositol (1,4,5)-Trisphosphate Biosynthesis	3.63E-03	2/27	PLCB3,PLCG1
NRF2-mediated Oxidative Stress Response	4.38E-03	4/199	CAT,DNAJB9,GSTA1,GSTM3
Glutathione-mediated Detoxification	4.76E-03	2/31	GSTA1,GSTM3
G Protein Signaling Mediated by Tubby	5.07E-03	2/32	PLCB3,PLCG1
**Top Networks**	**Score**	**#Focus Molecules**	**Focus Molecules**
Cardiovascular Disease, Organismal Injury and Abnormalities, Reproductive System Disease	56	24	ADRA2B,Akt,ALOX5AP,ATF6,ATP13A2,CAT,CELSR1,CYB5R4,DNAJB9,ERK,ERK1/2,Gpcr, GSTA1, HRH4,Insulin,Jnk,LRIG1,MAGI2,Mapk,NCF4,NFASC,NRCAM,NSMF,P38MAPK,PDIA6,Pkc(s), PLCB3, PLCG1,SNAP91,SRC(family),SRCIN1,TRPM4,Vegf,VRK2

Cell-to-Cell Signaling and Interaction, Nervous System Development and Function, Cardiovascular Disease	30	15	AAMDC,APP,C2orf49,CLSTN1,CYTOR,DNAJC4,EGFR,EML6,FBXO38,GLRB,GYS1, MAPK14, miR-124-3p(andothermiRNAsw/seedAAGGCAC),NEFH,PCDHGA3,PLEKHG2,RHBDF1,SERAC1, SGCG, SLC17A5,SNX13,SNX33,STX10,TENM1,TRIM32,TRIM45,TSEN2,TTC7A,Ttf1,UBE2E2, UGT2B10,USE1,ZNF449,ZNRF1
Cancer, Organismal Injury and Abnormalities , Respiratory Disease	23	12	ABCA4,AFMID,ARFGAP2,ATAD2,BCOR,DNAJC1,DNAJC21,DTNB,EIF4G1,EPRS,ESR1,FAM174A, FBXO34,Gsta4,GSTM3,HACD1,HEPHL1,HNRNPA1L2,HSP90AA1,HSPA13,JMY,MYO9B,PCNT, RPN2,SCFD1,SEC22A,SLFN1,SPG7,TCF25,TDRD9,TESK1,TSSK3,VCP,ZBED4
Cellular Assembly and Organization, Cell Morphology, Cellular Function and Maintenance	20	11	AP1G2,ATG,ATG7,ATG10,ATG13,ATG14,ATG101,ATG16L2,ATG2A,ATG2B,ATG4A,ATG4B, ATG4C, ATG4D,ATG9B,BSN,E2F1,FBXO46,FHDC1,HECTD4,ITM2B,MAP1LC3B2,Myh9l1,PCBP2,PNPLA8, PRICKLE3,RABEP1,RAD54L2,RUVBL1,TMEM59L,TXNL1,UBC,ZFYVE26,ZNF160
Developmental Disorder, Hereditary Disorder, Organismal Injury and Abnormalities	16	9	ADGRB3,BRINP2,CHD8,CLSTN3,CREB1,DNAH9,DNAH10,DYM,DYNLT1,FBXO22,FFAR3, GALR1 ,GHSR,GPR12,GPR19,GPR63,Hmgn2(includesothers),mir-28,mir-188,mir-296,mir-500,miR029b-3p(andothermiRNAsw/seedAGCACCA),MT1L,NEXMIF,P2RY11,SERINC5,SLC22A1,SMN1/SMN2, SSTR1,SZT2,TCF4,TP53,Zim1,ZPR1

Overlap: indicates the number of molecules in our dataset involved in a given pathway/the total number of known molecules in this pathway.
Focus molecule: are the network eligible molecules in our dataset. Score: is used to rank networks and is derived from the p-value which in turn calculates the probability that focus molecules are found in a given network from the Ingenuity® Knowledge base due to chance.

Supplementary Table S5. Over-represented functional categories from GO enrichment analysis.

GO_Term	Fold Enrichment	p-value	#Molecules	Molecules
GO_Biological process				
System development (GO:0048731)	2.18	2.22E-02	33	NSMF, SRCIN1, LRIG1, ATF6, NEFH, MAGI2, BSN, BRINP2, CLSTN3, FBXO38, CHD8, SGCG, GSTM3, DNAJB9, ITM2B, GLRB, CELSR1, CAT, NFASC, PLCG1, DYM, TENM1, ZNF160, ZPR1, GYS1, SPG7, BCOR, SZT2, NOTCH4, ADRA2B, EIF4G1, NRCAM, TRIM32
Nervous system development (GO:0007399)	3.01	4.02E-03	24	NSMF, SRCIN1, LRIG1, NEFH, MAGI2, BSN, BRINP2, CLSTN3, FBXO38, CHD8, GSTM3, ITM2B, GLRB, CELSR1, NFASC, PLCG1, TENM1, ZPR1, SPG7, SZT2, ADRA2B, EIF4G1, NRCAM, TRIM32
Unclassified	0.34	0.00E+00	4	FBXO46, DTNB, FAM174A, FBXO34
GO_Cellular component				
Membrane (GO:0016020)	1.53	2.28E-02	53	NSMF, SRCIN1, PRICKLE3, LRIG1, ATF6, PLCB3, ATG16L2, MAGI2, ATG2B, HEPHL1, CLSTN3, SNX33, TRPM4, SGCG, EPRS, SNAP91, CYB5R4, ZFYVE26, PCDHB8, DNAJB9, ITM2B, ALOX5AP, NCF4, RABEP1, VRK2, PCDHGA3, GLRB, CELSR1, CAT, NFASC ,PLCG1, CLSTN1, DYM, TENM1, CAT, SLC22A1, ATP13A2, GYS1, SPG7, SEC22A, SZT2, PDIA6, FAM174A, NOTCH4, ADRA2B, HRH4, HECTD4, PCNT, USE1, EIF4G1, UGT2B10, NRCAM, STX10
Cytoplasmic part (GO:0044444)	1.51	4.57E-02	52	NSMF, TSEN2, ATF6, PLCB3, NEFH, ATG16L2, MAGI2, BSN, BRINP2, ATG2B, CLSTN3, SNX33, TRPM4, USE1, ATG4C, EPRS, SNAP91, CAT, CYB5R4, ZFYVE26, FBXO22, GSTM3, DNAJB9, ITM2B, ALOX5AP, NCF4, RABEP1, VRK2, CAT, NFASC, PLCG1, CLSTN1, DYM, TENM1, CAT, ZPR1, ATP13A2, GYS1, GSTA1, SPG7, SEC22A, SZT2, PDIA6, NOTCH4, DNAH9, AFMID, PCNT, USE1, EIF4G1, UGT2B10, TRIM32, STX10

Neuron part (GO:0097458)	3.01	4.67E-02	17	NSMF, SRCIN1, NEFH, MAGI2, BSN, BRINP2, CLSTN3, TRPM4, GLRB, NFASC, CLSTN1, TENM1, ZPR1, ATP13A2, HRH4, NRCAM, STX10
Postsynaptic density (GO:0014069)	8.39	2.97E-02	7	NSMF, SRCIN1, NEFH, MAGI2, BSN, CLSTN3, CLSTN1
Postsynaptic specialization (GO:0099572)	8.32	3.14E-02	7	NSMF, SRCIN1, NEFH, MAGI2, BSN, CLSTN3, CLSTN1
Asymmetric synapse (GO:0032279)	8.22	3.40E-02	7	NSMF, SRCIN1, NEFH, MAGI2, BSN, CLSTN3, CLSTN1
Neuron to neuron synapse (GO:0098984)	8.08	3.78E-02	7	NSMF, SRCIN1, NEFH, MAGI2, BSN, CLSTN3, CLSTN1
Unclassified	0.36	0.00E+00	3	FBXO46, FBXO34, UBE2E2
GO_Molecular Function				
Unclassified				

Supplementary Table S6. Brain specific expression in different databases of genes identified In this study.

Gene	Expression databases	Basal ganglion	Brain	Cerebral cortex	Hippocampus	Midbrain	Substantia nigra
NOTCH4	68 FANTOM5 project - adult	NA	+	NA	NA	NA	+
	32 Uhlen's Lab	NA	NA	+	NA	NA	NA
ADRA2B	68 FANTOM5 project - adult	NA	NA	NA	NA	NA	NA
	32 Uhlen's Lab	NA	NA	-	NA	NA	NA
ATP13A2	68 FANTOM5 project - adult	NA	++	NA	NA	NA	++
	32 Uhlen's Lab	NA	NA	++	NA	NA	NA
CELSR1	68 FANTOM5 project - adult	NA	-	NA	NA	NA	-
	32 Uhlen's Lab	NA	NA	-	NA	NA	NA
CLSTN3	68 FANTOM5 project - adult	NA	+	NA	NA	NA	0
	32 Uhlen's Lab	NA	NA	++	NA	NA	NA
DYM	68 FANTOM5 project - adult	NA	++	NA	NA	NA	++
	32 Uhlen's Lab	NA	NA	+	NA	NA	NA
EIF4G1	68 FANTOM5 project - adult	NA	++	NA	NA	NA	+
	32 Uhlen's Lab	NA	NA	++	NA	NA	NA
EPRS	68 FANTOM5 project - adult	NA	+	NA	NA	NA	+
	32 Uhlen's Lab	NA	NA	++	NA	NA	NA

FAM174A	68 FANTOM5 project - adult	NA	++	NA	NA	NA	++
	32 Uhlen's Lab	NA	NA	++	NA	NA	NA
HEPHL1	68 FANTOM5 project - adult	NA	-	NA	NA	NA	-
	32 Uhlen's Lab	NA	NA	-	NA	NA	NA
HRH4	68 FANTOM5 project - adult	NA	NA	NA	NA	NA	NA
	32 Uhlen's Lab	NA	NA	-	No data	NA	No data
MAGI2	68 FANTOM5 project - adult	NA	+	NA	NA	NA	+
	32 Uhlen's Lab	NA	NA	++	NA	NA	NA
NEFH	68 FANTOM5 project - adult	NA	++	NA	NA	NA	++
	32 Uhlen's Lab	NA	NA	++	NA	NA	NA
PLCB3	68 FANTOM5 project - adult	NA	+	NA	NA	NA	+
	32 Uhlen's Lab	NA	NA	+	NA	NA	NA
PUM1	68 FANTOM5 project - adult	NA	++	NA	NA	NA	++
	32 Uhlen's Lab	NA	NA	++	NA	NA	NA
SNAP91	68 FANTOM5 project - adult	NA	+	NA	NA	NA	-
	32 Uhlen's Lab	NA	NA	++	NA	NA	NA
BCOR	68 FANTOM5 project - adult	NA	-	NA	-	NA	-
	32 Uhlen's Lab	NA	NA	++	NA	NA	NA
CLSTN1	68 FANTOM5 project - adult	NA	+	NA	+	NA	-
	32 Uhlen's Lab	NA	NA	++	NA	NA	NA

ITM2B	68 FANTOM5 project - adult	NA	++	NA	++	NA	++
	32 Uhlen's Lab	NA	NA	++	NA	NA	NA

(-) Expression level is below cutoff (0.5 FPKM).
(+) Expression level is low (between 0.5 to 10 FPKM).
(++) Expression level is medium (between 11 to 1000 FPKM).
(+++) Expression level is high (more than 1000 FPKM).
(NA) There is no data available.

Supplementary Table S7. Identified variants present in the SHGP pandb.

Identified Variants		SHGP pandb			ClinVar	
**Gene**	**Variant**	**Genotype**	**Total cases**	**Phenotype (#cases)**	**ID**	**Classification**
ADRA2B	p.R222X	Homozygous	1	Neurodegeneration	191117	Likely pathogenic
NOTCH4	c.2865+2T>C	Homozygous	1	Neurodegeneration	NA	NA
PLCB3	c.698+4A>T	Heterozygous	3	Neurodegeneration (2)<br>Ataxia (1)	NA	NA
BCOR	p.V160I	Heterozygous (female)	1	Neurodegeneration	NA	NA
EIF4G1	p.K715R	Heterozygous	7	Neuropathy (5)<br>Ataxia (1)<br>Ataxia and Neurodegeneration (1)	NA	NA
MAGI2	c.1280C>T	Heterozygous	1	Neurodegeneration	NA	NA
CLSTN1	p.Q185E	Heterozygous	1	Neurodegeneration	NA	NA

NA: Not listed in ClinVar.(https://www.ncbi.nlm.nih.gov/clinvar/)

Supplementary Table S8. Mouse models with neurological and behavioural features

Gene	Mouse model(s) (MGI Genotype _ID)	Mouse model of Human Disease (DO_ ID/MGI Genotype _ID)	Mouse models with neurological/ Behavioural abnormalities	Neurological/Behavioural Features	Shared features
Celsr1	1.MGI:5661735<br>2.MGI:2668352<br>3.MGI:5661738<br>4.MGI:4948448<br>5.MGI:2668349<br>6.MGI:3690972<br>7.MGI:5751481<br>8.MGI:5751522<br>9.MGI:2668355<br>10.MGI:2174732<br>11.MGI:2668356<br>12.MGI:4430219<br>13.MGI:4430220<br>14.MGI:4949261<br>15.MGI:4949260	NA	1.MGI:5661735<br>2.MGI:2668352<br>3.MGI:5661738<br>4.MGI:2668349<br>5.MGI:3690972<br>6.MGI:5751481<br>7.MGI:5751522<br>8.MGI:2668355<br>9.MGI:2174732<br>10.MGI:2668356<br>11.MGI:4430219	delayed neural tube closure<br>abnormal neural plate morphology<br>head shaking<br>spinning<br>craniorachischisis<br>abnormal orientation of outer hair cell stereociliary bundles<br>incomplete rostral neuropore closure<br>circling<br>head tossing<br>hydroencephaly<br>cochlear outer hair cell degeneration<br>abnormal involuntary movement<br>head bobbing<br>abnormal outer hair cell kinocilium morphology<br>incomplete rostral neuropore closure	Stereotypic behaviours<br>Neural tube defects<br>Vestibular system defects

Bsn	1.MGI:4880011<br>2.MGI:2652947	NA	1.MGI:4880011<br>2.MGI:2652947	abnormal inner hair cell synaptic ribbon morphology<br>abnormal brain wave pattern<br>abnormal CNS synaptic transmission<br>abnormal cochlear inner hair cell physiology<br>abnormal retinal rod cell morphology<br>abnormal somatic nervous system physiology<br>abnormal synaptic transmission<br>abnormal synaptic vesicle number<br>absent active-zone-anchored inner hair cell synaptic ribbon<br>ataxia<br>clonic seizures<br>convulsive seizures<br>decreased cochlear nerve compound action potential<br>decreased synaptic depression<br>impaired righting response<br>increased susceptibility to pharmacologically<br>induced seizures myoclonus	abnormal inner hair cell synaptic ribbon morphology

Snap91	MGI:5811248	NA	1MGI:5811248	abnormal brain wave pattern<br>abnormal CNS synaptic transmission<br>abnormal excitatory postsynaptic potential<br>abnormal excitatory synapse morphology<br>abnormal inhibitory postsynaptic currents<br>abnormal inhibitory synapse morphology<br>abnormal miniature excitatory postsynaptic currents<br>abnormal nest building behaviour<br>abnormal response to novel object<br>abnormal synaptic vesicle number<br>abnormal synaptic vesicle morphologyplasticity<br>abnormal synaptic vesicle recycling<br>decreased anxiety-related response<br>decreased excitatory postsynaptic current amplitude<br>enhanced paired-pulse facilitation<br>hyperactivity<br>limb grasping<br>tonic-clonic seizures	NA
Spg7	MGI:3028926	hereditary spastic paraplegia 7 (0110816/3028926)	MGI:3028926	abnormal axon morphology<br>abnormal axonal transport<br>abnormal motor coordination/ balance<br>axon degeneration<br>impaired balance<br>impaired coordination<br>impaired limb coordination	NA

Magi2	1.MGI:5660913<br>2.MGI:5688181<br>3.MGI:5688180<br>4.MGI:5688182<br>5.MGI:3716616<br>6.MGI:5620953	schizophrenia (5419/5620953)	1.MGI:3716616<br>2.MGI:5620953	abnormal neuron morphology<br>abnormal dendritic spine morphology<br>abnormal excitatory postsynaptic potential<br>abnormal nest building behavior<br>abnormal social investigation<br>abnormal social/conspecific interaction<br>abnormal spatial working memory<br>anhedonia<br>decreased prepulse inhibition<br>enlarged lateral ventricles<br>hyperactivity<br>increased anxiety-related response<br>reduced long term potentiation	abnormal neuron morphology
Atp13a1	1.MGI:5642335<br>2.MGI:5812991<br>3.MGI:5472145	Kufor-Rakeb syndrome (0060556/5642335)	1.MGI:5642335<br>2.MGI:5472145	abnormal neuron morphology<br>astrocytosis<br>hypoactivity<br>limb grasping<br>abnormal gait<br>abnormal motor coordination/ balance<br>abnormal nest building behaviour<br>abnormal response to novel object<br>abnormal spatial learning<br>alpha-synuclein inclusion body<br>short stride length	abnormal neuron morphology
Eprs	1.MGI:5767654<br>2.MGI:5757056	NA	MGI:5767654	abnormal forebrain development<br>abnormal hindbrain development<br>abnormal midbrain development<br>abnormal neural tube closure<br>abnormal spinal cord morphology	NA
Dnajb9	1.MGI:5699098<br>2.MGI:5699100<br>3.MGI:5699101	NA	1.MGI:5699098<br>2.MGI:5699100	circling<br>head tilt<br>hyperactivity	Stereotypic behaviours<br>Hyperactivity

Clstn3	1.MGI:5523436<br>2.MGI:5756884	NA	1.MGI:5523436<br>2.MGI:5756884	abnormal dentate gyrus morphology<br>abnormal excitatory postsynaptic potential<br>abnormal hippocampus CA1 region morphology<br>abnormal hippocampus pyramidal cell layer<br>abnormal hippocampus stratum radiatum morphology<br>abnormal miniature excitatory postsynaptic currents<br>abnormal gait<br>tremors	NA
Nefh	1.MGI:3694631<br>2.MGI:3694558<br>3.MGI:3851998<br>4.MGI:3842789<br>5 MGI:4947054<br>6.MGI:2183678<br>7.MGI:2183672<br>8.MGI:4947056<br>9.MGI:4947060<br>10.MGI:4947061<br>11.MGI:2183668<br>12.MGI:2183662	amyotrophic lateral sclerosis type 1 (0060193/2183678,2183672, 2183668 , 2183662)	1.MGI:3694631<br>2.MGI:3694558<br>3.MGI:3851998<br>4.MGI:3842789<br>5 MGI:4947054<br>6.MGI:2183678<br>7.MGI:2183672<br>8.MGI:4947056<br>9.MGI:4947060<br>10.MGI:4947061<br>11.MGI:2183668<br>12.MGI:2183662	abnormal axonal transport<br>abnormal axon morphology<br>abnormal neuron morphology<br>abnormal ventral spinal root morphology<br>decreased motor neuron number<br>axon degeneration<br>hindlimb paralysis<br>abnormal action potential<br>abnormal motor neuron morphology<br>limb grasping<br>motor neuron degeneration<br>tremors<br>decreased sensory neuron number<br>paralysis	abnormal axon morphology (7 models)<br>motor neuron degeneration (5 models)<br>paralysis (2)<br>abnormal motor neuron morphology (6)<br>limb grasping (6)<br>tremors (6)
Notch4	1.MGI:5790679<br>2.MGI:5790678<br>3.MGI:5304927<br>4.MGI:5797682<br>5.MGI:5790676<br>6.MGI:2175161<br>7.MGI:5790675<br>8.MGI:3720608<br>9.MGI:5502689	Arteriovenous Malformations of the brain (0060688/5502689)	MGI:5502689	abnormal brain morphology<br>abnormal brain vasculature morphology<br>cerebral arteriovenous malformation<br>abnormal midbrain morphology<br>abnormal neocortex morphology<br>abnormal cerebellum morphology<br>abnormal nervous system physiology<br>cerebellum hemorrhage<br>intraventricular hemorrhage<br>increased neuron apoptosis<br>ataxia<br>seizures	NA

Itm2b	MGI:3790078<br>MGI:6149954<br>MGI:3810987<br>MGI:3810986<br>MGI:4415610<br>MGI:4867871	cerebral amyloid angiopathy	MGI:3810987<br>MGI:4415610<br>MGI:4867871	impaired contextual conditioning behaviour<br>abnormal object recognition memory<br>abnormal long term object recognition memory<br>abnormal spatial working memory<br>reduced long term potentiation<br>behaviour/neurological phenotype<br>impaired contextual conditioning behaviour<br>abnormal object recognition memory<br>abnormal spatial working memory<br>nervous system phenotype<br>reduced long term potentiation<br>impaired contextual conditioning behaviour<br>abnormal long term object recognition memory<br>abnormal spatial working memory	impaired contextual conditioning behaviour<br>abnormal object recognition memory<br>abnormal spatial working memory
Clstn1	MGI:3530091<br>MGI:5637427<br>MGI:4950238<br>MGI:5548714<br>MGI:5637426<br>MGI:2674107		MGI:5637427<br>MGI:5548714	abnormal dendrite morphology<br>abnormal dendritic spine morphology<br>abnormal CNS synaptic transmission<br>abnormal excitatory postsynaptic currents<br>prolonged excitatory postsynaptic current decay time<br>abnormal excitatory postsynaptic potential<br>abnormal glutamate-mediated receptor currents<br>abnormal NMDA-mediated synaptic currents<br>enhanced long term potentiation<br>abnormal locomotor activation<br>straub tail	NA

Only the mouse models of the prioritized genes in main table 1 are listed here.

Supplementary Table S9. Summary of clinical data

						Non-motor symptoms		
**Patient**	**Age (years)**	**Gender**	**Smoking (Y/N)**	**Family history**	**Movement**	**Memory/Cognitive/Speech**	**Mood/Emotional/ Psychological/Sleep**	**Other Medical Conditions**
PD-1	64	M	NA	NA	Rigidity, dyskinesia, recurrent falls and freezing of gait	Patient has adversely affected language functions (slurred speech) as a result of a stroke. Also, has a severe hypokinetic dysarthria along with severe neurogenic stuttering with limited response to intervention	Depression	Dysphagia, venous infarct over the left temporal lobe.
PD-2	53	M	NA	NA	Bradykinesia, tremor, stiffness, decreased range of motion and gait abnormality	Mild softening of the speech volume	NA	NA
PD-3	NA	NA	NA	NA	Tremors	NA	NA	G6PD, ECG showed prolonged QT interval
PD-4	55	M	N	NA	Sudden speed causing falls	NA	NA	IDDM, Acute myocardial infarction of inferior wall
PD-5	64	M	NA	NA	Bradykinesia, rigidity tremor, shuffling gait, mask-like expression, hypertonic dystonia (left-side).<br>Cogwheel rigidity and bilateral brisk reflexes with no associated cerebellar signs.<br>The patient has bilateral decreased blinking with no signs of extraocular movement abnormalities	NA	NA	Duodopa device<br>Bedside bilateral Botox injection of parotid glands for increased salivation
PD-6	83	M	N	NA	Frequent falls	NA	NA	HTN, DM, benign prostatic hyperplasia, metastatic colon cancer and renal failure
PD-7	68	M	NA	NA	NA	NA	NA	HTN, NIDDM
PD-8	76	M	NA	NA	Tremors (affecting the right more than the left side), poor balance,	NA	Experience bad dreams and occasional hallucinations	HTN, urinary urgency and cconstipation

					mask-like expression (affecting the right more than the left side) with moderate bradykinesia, rigidity and normal postural reflexes			
PD-10	51	M	NA	Positive family history of PD	Dystonia of the right foot, motor fluctuation and sever dyskinesia. Patient had good response to Duodopa infusion pump.	Disturbed speech	NA	NA
PD-11	75	M	N	Positive family history	Right leg tremor, upper and lower limb rigidity and spasticity. Patient received deep brain stimulation	NA	NA	Right indirect inguinal hernia and benign prostate hyperplasia
PD-12	40	M	NA	NA	Mild on and off spasticity of the leg for which was investigated for anti-GAD antibodies and was negative.	NA	NA	Deltoid muscle biopsy was done and electro-microscopic showed abnormal mitochondria suggestive of mitochondrial disease. High lactic acid and high pyruvic acid on several occasions. Mitochondrial myopathy. Type I DM.
PD-13	73	M	NA	NA	NA	NA	NA	DM, HTN, and dyslipidemia
PD-14	65	M	NA	Mother affected with early onset-PD (diagnosed at 50 years of age)	Resting tremor in the left arm, mild cogwheel rigidity, bradykinesia and hypokinesia mainly on the left side, decreased arm swinging with slightly stooped posture and some start hesitation. Patient received deep brain stimulation with good response.	NA	Tendency for impulse control problem	NA
PD-15	23	M	NA	NA	Non-ambulatory (on a wheel chair) and walks with assistance, has frequent attacks of	NA	Experience vivid dream, visual and auditory hallucinations	Constipation, salivation urinary incontinence,

					dystonia and dysphagia. The patient received Dudopa infusion pump insertion.			
PD-16	72	F	NA	3 affected siblings, affected uncle and aunt	Bradykinesia, asymmetrical tremors, difficulty in standing and walking, bilateral rigidity and loss of postural reflexes. Peak dose dyskinesia was reported.	NA	Tendency for depression and experiencing bad dreams.	slightly broken saccadic eye movement
PD-17	63	F	NA	NA	Mild intention tremor affecting the right side more than the left side with impaired postural reflexes	NA	NA	NA
PD-18	42	F	NA	Deceased grandfather had similar symptoms	Bradykinesia, action tremor started in the upper right limb then progressed to involve the left arm and abnormal posturing of limbs	Cognitive symptoms were reported (increased forgetfulness)	Hallucinations	NA
PD-19	73	M	NA	Affected brother and mother	Bradykinesia, postural instability with peak-dose dyskinesia and end-of-dose deterioration. Patient received deep brain stimulation.	NA	Impulse control disorder, clear paranoia and nocturnal hallucination, REM related behavior problem and signs of depression	NA
PD-20	61	M	NA	Affected brother and mother	NA	NA	NA	NA
PD-21	56	M	NA	Positive family history	Patient had peak-dose dyskinesia and was given Duodopa infusion pump.	NA	Depressive disorder with some persistent psychotic features	
PD-23	38	M	NA	NA	Bradykinesia, impaired posture and gait problem. Patient had peak-dose dyskinesia therefore her received deep brain stimulation	NA	Anxiety, lack of sleep with restless leg and arm movements and talking during sleep,<br>low mood and continuous fatigue	
PD-24	70	M	NA	NA	Rigidity, tremor dysphagia	Some signs of cognitive impairment (amnesia)	Impulsivity and aggressiveness,	HTN, sweating, urine retention, seizure attacks and incontinence

							REM related behavior problems with visual hallucinations	
PD-26	82-deceased	F	NA	NA	Tremors, rigidity dyskinesia	Slurred speech	NA	DM, HTN, atrial fibrillation, and decompensated heart failure
PD-28	33	M	NA	NA	Non-ambulatory (on a wheel chair) and	NA	NA	Autoimmune hyperthyroidism, psoriasis, scoliosis, influenza B, and thyrotoxicosis
PD-29	72	M	N	NA	Rigidity, dyskinesia and motor fluctuations	some signs of cognitive impairment, worsening dysarthria	NA	NA
PD-30	82	M	NA	NA	Rigidity, tremors, start hesitation and recurrent falls	NA	NA	Osteoarthritis, fractures, adenocarcinoma, HTN, incontinence, DM, osteoporosis, bilateral hip replacement, prostate cancer, right shoulder lipoma and thoracic spinal cord injury
PD-31	60	M	NA	NA	NA	NA	NA	NA
PD-32	63	M	NA	Sister with similar symptoms	Tremors, bradykinesia freezing gait, and mask-like expression	Dysarthria	Sleep problems	DM type 2 and HTN
PD-33	69	M	N	NA	NA	NA	NA	NA
PD-34	72	M	NA	Son has similar tremors	Bilateral long standing resting tremors, late onset bradykinesia and rigidity. Patient had poor response to typical PD treatments including deep brain stimulation	NA	NA	Cataract
PD-35	56	M	N	NA	Postural instability and mask-like expression. Patient received deep brain stimulation	Dysarthria and memory impairment	Depression	thrombocythemia, transient ischemic attacks, peptic ulcer, myelofibrosis and cystic kidney disease
PD-36	77	M	NA	NA	Tremor, rigidity, bradykinesia, postural	NA	NA	HTN

					instability and slowed gait. Occasional peak-dose dyskinesia was reported. Patient not compliant with anti-Parkinson medication			
PD-37	44	M	N	NA	Tremor and bradykinesia. Occasional increase in speed of gait when on Sinemet that causes the patient to hit the wall, therefore patient underwent deep brain stimulation.	NA	NA	NA
PD-39	66	M	NA	NA	NA	NA	NA	NA
PD-40	54	M	NA	NA	Rigidity, bradykinesia, resting tremor and mask-like expression	NA	possible signs of depression	NA
PD-41	55	M	NA	NA	Tremor, dyskinesia mild to moderate rigidity and repeated falls. Patient underwent deep brain stimulation	NA	Visual hallucinations	DM, HTN, urinary incontinence, dilated cardiomyopathy, and supraventricular tachycardia
PD-42	68	M	N	No family history of idiopathic Parkinson disease	Mask-like expression, resting tremor, head tremor akinesia, bradykinesia, and rigidity. Suffers from dysphagia and weight loss. Patient is Non-ambulatory (on a wheel chair), feels dizzy when standing up from sitting position and has frequent falls.	Problems with speech in the form of dysphonia	Sometime has difficulty sleeping at night	Cervical spondylosis with degenerative disc at C3-C4, C4-C5, C5-C6 and C6-7 levels, urinary incontinence, constipation, optical impression-R and corneal opacity with initial lens changes are positive
PD-43	67	M	NA	NA	Left hand tremors	NA	NA	IDDM
PD-44	44	F	NA	NA	NA	NA	NA	NA
PD-45	69	M	NA	early onset, slow progression	Significant dyskinesia (neck and extremities) and impaired postural reflexes. The patient started showing	NA	NA	NA

					asymmetrical tremors at the age of 49 years			
PD-46	30	F	NA	family history of PD, father and father's sister	Tremor of both hands	NA	NA	NA
PD-47	53	M	N	NA	Right arm bradykinesia, mild motor fluctuation with peak dose dyskinesia	NA	Suicidal thoughts with fragmented sleep and sometime vivid dreams	NA
PD-48	55	F	NA	NA	Dyskinesia, bilateral resting tremor, mask-like expression, cogwheel rigidity and abnormal ataxic-like gait. Duodopa infusion pump insertion triggered peak-dose dyskinesia with increased slowness, rigidity, freezing, and frequent falls.	Decreased communication in the family	Visual hallucination, sleep disturbance and insomnia.<br>Panic attacks.<br>Decreased appetite.	Bilateral DVT and bed sores
PD-49	80	M	NA	Positive family history	NA	NA	NA	NA
PD-50	73	F	NA	Positive similar family history in two brothers	Bradykinesia, dyskinesia, dystonic posturing of the right leg, bilateral resting tremor (right leg), cogwheel rigidity<br>(left more than right side) and<br>shuffling gait with short steps	NA	NA	Corneal ring not typical for Kayser-Fleischer Ring.
PD-52	68	F	N	NA	Fall and had fracture of the left fibula	NA	Mood swings with bad tempers at times	DM, HTN, ischemic heart disease, morbid foot and leg edema, osteoarthritis and nocturnal dyspnea
PD-53	75	F	N	NA	Rigidity and dystonia involving all limbs	Dementia Rating Scale-2: attention score (39), initiation/perseveration score (8), constructional abilities score (5), conceptualization score (32), memory score (25). Dementia Rating Scale total	NA	Poor hearing acuity and HTN

						score (109). Mini mental state examination score of (21).		
PD-54	67	M	ex-smoker for 20 years	NA	Unilateral tremors and bradykinesia (right side), mild dyskinesia affecting the neck and right upper extremity and tendency to fall	NA	Depression	HTN and DM
PD-55	83	M	NA	NA	NA	NA	NA	NA
PD-56	65	M	NA	NA	Dyskinesia and postural impairment with peak-dose dyskinesia	NA	NA	NA
PD-57	49	F	NA	NA	Bradykinesia, postural instability, impaired gait and cogwheel rigidity (left side more severe than the right). Mild-to-moderate peak dose dyskinesia was reported	Monotonic speech	NA	NA
PD-58	35	M	NA	NA	Tremors, mild rigidity and dyskinesia. Patient received Duodopa infusion pump.	NA	NA	NA
PD-60	78	M	N	NA	Bradykinesia	NA	NA	IDDM, ischemic heart disease and anterior myocardial infarction
PD-62	58	M	NA	NA	Unilateral (right side) tremor, poor motor control and bradykinesia	NA	Vivid dreams, and night No frank hallucination during the day	Pallidotomy
PD-63	69	M	NA	NA	Bradykinesia, tremors, rigidity, postural impairment. Patient is Non-ambulatory (on a wheel chair).	Dementia	REM related behavior problems (night hallucinations and vivid dreams)	DM, HTN, CAD, stroke, chronic iron deficiency and anemia,
PD-64	46	M	N	NA	Bradykinesia and dyskinesia. Peak-dose and low-dose dyskinesia were reported.	NA	NA	DM type 2 and hyposmia
PD-76	81	M	NA	NA	Mild rigidity, mask-like expression, start hesitation and dysphagia.	Dysarthria and dysphonia	Mild depression	Cardiac problems and hypercholesterolemia

PD-77	59	M	NA	NA	Mask-like expression, bilateral bradykinesia in upper and lower limbs with cogwheel rigidity.	Could recall 3 out of 4 objects with little difficulty. Speech is slow and metonymic.	NA	Back and lower extremity pain
PD-78	85	M	NA	NA	Non-ambulatory (on a wheel chair), bilateral cogwheel rigidity, and paratonia	Can follow simple commands and recalled 2/4 objects with difficulty. Speech is very slow and faint	Vivid dreams, nocturnal hallucination, and psychiatric problems	Arthritis and bilateral visual impairment.
PD-79	75	M	NA	NA	Bradykinesia with frequent falls and slow progression. Patient experience occasional choking to thin liquids only.	NA	Screams	DM, HTN, ischemic heart disease orthostatic hypotension, osteoporosis, urinary frequency and nocturia. Patient underwent heart stenting and right hip replacement procedures.

HTN: Hypertension, DM and IDDM: Diabetes Mellitus and Insulin Dependent Diabetes Mellitus, CAD: Coronary Artery Disease, DVT: Deep Vein Thrombosis, G6PD: G6PD deficiency, NA: Information not available. Age: patient age at the time of drafting the manuscript.

Supplementary Table S10. Summary Of Brain Imaging Data.

Patient	Imaging summary
PD-1	TL: Encephalomalacic changes noted in the left + small old hemorrhage. Brain stem: T2 hyperintensity bilaterally. Cerebellum: scattered foci of blooming. + Mild volume loss involves the superior cerebellar vermis. Whole brain: Confluent and patchy T2 hyperintensities present in the cerebral WM bilaterally. Other: T2 hyperintensity noted in the left sigmoid sinus.
PD-2	C-spine revealed reversal of cervical lordosis with decreased pulposus of the annulus fibrosus at C5/6.
PD-3	Global central and cortical volume loss. The pituitary stalk is possibly very diminutive and posteriorly displaced within the sella turcica. CSF: prominence of the intra and extra-axial CSF spaces.
PD-4	SN: slightly thinner on the right side. TL: encephalomalacic changes in the left + old hemorrhage likely secondary to prior hemorrhagic venous infarction. Other: T2 hyperintensity in the left sigmoid sinus (chronic thrombus).
PD-5	Periventricular Changes: CT / small ill-defined frontal periventricular low attenuation focus and a second questioned similar abnormality in the frontal centrum semiovale periventricular region. Whole brain: CT/ The GM/WM differentiation is otherwise well maintained.
PD-11	Parenchyma demonstrates normal signal intensity.
PD-12	PL: In the left parietal region there are small extraaxial flow voids identified that are adjacent to the calvarium, probably in the subdural space with minimal enhancement (small dural arteriovenous malformation). Other: Sinusitis involving the left maxillary sinus and to a lesser extent the ethmoid sinuses.
PD-15	FL: bilateral moderate atrophy. TL: bilateral moderate medial lobe atrophy. Cortex: bilateral moderate cerebral cortical atrophy. Caudate nucleus: volume loss. CSF: widening ventricular system. Other: mildly enlarged nasopharyngeal lymphoid tissue.
PD- 18	Normal MRI.
PD-19	CT/ DBS probe.
PD- 23	CT/ DBS electrodes are frontal. Bilateral degenerative calcification of the palatine tonsils and small nonsignificant right maxillary sinus floor retention cyst.
PD- 24	Periventricular changes: CT/occipital horn intraventricular hemorrhage. FL: CT/bilateral frontal polar nondependent tension pneumocephalus with mild distorted mass effect on subjacent parenchyma. Cortex: CT/pneumocephalus along the cortical portion of the right electrode. Other: CT/ asymmetric prominence of the left side cisternal trigeminal nerve worrisome for trigeminal nerve abnormality.
PD- 26	Periventricular WM bright T2/FLAIR signal intensity indicating old microangiopathic ischemic insult. Brain stem: mildly decreased attenuation in the left cranial portion of the pons. Whole brain: Central volume loss. CSF: prominent ventricles.
PD-28	CST: high signal intensity in the corticospinal tracts up to the mesencephalon bilaterally and symmetrically. Pituitary gland: marked atrophy and high intensity signal in the inferior rim of pituitary gland. Other: significant parenchymal atrophy is seen supratentorially as well as infratentorially. CT/ There is polypoid mucosal disease in the bilateral maxillary sinuses and mucosal thickening in the left nasal cavity. Other:? ALS (suspicion).
PD- 29	Periventricular Changes: CT/asymmetric patchy periventricular WM low attenuation changes of probable small vessel disease. Other: CT/carotid and vertebral artery atherosclerotic calcifications.
PD- 30	CST: mild prominence of the cortical sulci. Cerebellum: cerebellar atrophy. Other: left sphenoid sinus demonstrates mild opacification with low intensity area filling the sphenoid sinus. CT/ show fractures.
PD- 32	Multiple small T2 hyperintense lesions are seen bilaterally. Other: ischemic lesions.
PD- 34	CT/Multiple low attenuated areas seen in the left subinsular and peritrigonal region on both sides as before. CST: CT/There is ill-defined focal hypodensity noted in the left corona radiata and subinsular region, likely related to an old ischemic insult.
PD- 35	CT/unremarkable.
PD- 37	FL: focal T2 hyperintense lesion. CT/unremarkable.
PD- 41	Unremarkable.
PD- 42	Whole brain: mild parenchymal atrophy supratentorially. Unremarkable.
PD- 44	Periventricular Changes: CT/asymmetric and predominantly frontal periventricular WM low-attenuation changes. CST: CT/low-attenuation changes left side internal capsule anterior limb. ? Vasculitis - ischemic -. Pituitary G.: CT/partially empty - nonexpanded sella turcica. Thalamus: CT/right side thalamic small focal low-attenuation. Other: CT/bilateral palatine tonsil degenerative dystrophic calcification. There is bilateral carotid artery calcifications of possible atherosclerotic nature.
PD- 52	Periventricular Changes: high signal intensity changes distributed bilaterally (? small vessel origin). Brain stem: mild general substance reduction and scattered lacunar infarctions in the basal ganglia on

	both sides. Cerebellum: Few high signal intensity changes distributed right side. Whole brain: few ischemic WM lesions. Other: moderate small vessel disease and a few scattered basal ganglia lacunar infarctions.
PD- 53	CT/unremarkable.
PD- 54	FL: mild frontoparietal atrophy. PL: mild frontoparietal atrophy.
PD- 55	Whole brain: Moderate parenchymal atrophy supratentorially.
PD- 57	FL: Bilateral mild volume loss. Two tiny frontal WM nonspecific hyperintense foci. SN: susceptibility signal of the red nucleus and SN.
PD-60	Left mastoid air cells.
PD-62	CT- Nonspecific involutional changes.
PD-63	Periventricular Changes: T2 hyperintensity. Cerebellum: Atrophy in the superior vermis. Whole brain: Decreased. scattered foci of T2 high signal intensity noted in WM
PD-64	CSF spaces are deeper than expected. Mild parenchymal atrophy supratentorially.
PD-79	Global volume loss. Periventricular changes: patchy WM T2 hyperintensity. PL: parietooccipital old tissue loss with marginal gliosis. Arachnoid cyst. CT/ Encephalomalacia in the left. CSF: prominence of the intra- and extraaxial CSF spaces with disproportionately larger prominence of the intraaxial CSF spaces> relative ballooning of the third ventricle and exaggerated CSF flow through the sylvian aqueduct. Other: minimal mucosal thickening of the paranasal sinuses.

BS: Brain Stem, WM: White Matter, GM: Grey Matter, CSF: Cerebrospinal Fluid, SN: Substantia Nigra, CST: Corticospinal Tract, PL: Parietal Lobe
TL: Temporal Lobe, FL: Frontal Lobe.

References

1. Al-Mubarak, B.R. *et al.* Parkinson's Disease in Saudi Patients: A Genetic Study. *PLoS One* **10**, e0135950 (2015).
2. Sun, M. *et al.* Influence of heterozygosity for parkin mutation on onset age in familial Parkinson disease: the GenePD study. *Arch Neurol* **63**, 826-32 (2006).
